# Elucidating Novel Targets for Ovarian Cancer Antibody–Drug Conjugate Development: Integrating In Silico Prediction and Surface Plasmon Resonance to Identify Targets with Enhanced Antibody Internalization Capacity

**DOI:** 10.3390/antib12040065

**Published:** 2023-10-16

**Authors:** Emenike Kenechi Onyido, David James, Jezabel Garcia-Parra, John Sinfield, Anna Moberg, Zoe Coombes, Jenny Worthington, Nicole Williams, Lewis Webb Francis, Robert Steven Conlan, Deyarina Gonzalez

**Affiliations:** 1 Reproductive Biology and Gynaecological Oncology Group, Swansea University Medical School, Swansea University, Singleton Park, Swansea SA2 8PP, UKd.w.james@swansea.ac.uk (D.J.); j.garciaparra@swansea.ac.uk (J.G.-P.); z.coombes@swansea.ac.uk (Z.C.); l.francis@swansea.ac.uk (L.W.F.); r.s.conlan@swansea.ac.uk (R.S.C.); 2 Cytiva, Björkgatan 30, 751 84 Uppsala, Sweden; john.sinfield@cytiva.com (J.S.); anna.moberg@cytiva.com (A.M.); 3 Axis Bioservices Ltd., 189 Castleroe Rd, Coleraine BT51 3RP, UK; jenny@axisbio.co.uk (J.W.); nicole@axisbio.co.uk (N.W.)

**Keywords:** ovarian cancer, antibody–drug conjugates, bioinformatics, in silico, biomarkers, therapeutics, internalization, SPR, Biacore

## Abstract

Antibody–drug conjugates (ADCs) constitute a rapidly expanding category of biopharmaceuticals that are reshaping the landscape of targeted chemotherapy. The meticulous process of selecting therapeutic targets, aided by specific monoclonal antibodies’ high specificity for binding to designated antigenic epitopes, is pivotal in ADC research and development. Despite ADCs’ intrinsic ability to differentiate between healthy and cancerous cells, developmental challenges persist. In this study, we present a rationalized pipeline encompassing the initial phases of the ADC development, including target identification and validation. Leveraging an in-house, computationally constructed ADC target database, termed ADC Target Vault, we identified a set of novel ovarian cancer targets. We effectively demonstrate the efficacy of Surface Plasmon Resonance (SPR) technology and in vitro models as predictive tools, expediting the selection and validation of targets as ADC candidates for ovarian cancer therapy. Our analysis reveals three novel robust antibody/target pairs with strong binding and favourable antibody internalization rates in both wild-type and cisplatin-resistant ovarian cancer cell lines. This approach enhances ADC development and offers a comprehensive method for assessing target/antibody combinations and pre-payload conjugation biological activity. Additionally, the strategy establishes a robust platform for high-throughput screening of potential ovarian cancer ADC targets, an approach that is equally applicable to other cancer types.

## 1. Introduction

Ovarian cancer (OC) is the leading cause of death from gynaecological malignancies. OC is usually diagnosed at an advanced stage since early symptoms are vague and there remains a lack of effective early-stage biomarkers to detect the disease [1]. Less than half of women with OC survive five years from diagnosis, with many unresponsive to current treatments [2]. Cytoreductive surgery and platinum- and taxane-based chemotherapy are the first-line treatment options for ovarian carcinoma. Unfortunately, despite a positive initial response to chemotherapy, 70–80% of patients with advanced stage OC subsequently develop recurrent disease, and about half of these patients eventually develop platinum resistance, leading to an unfavourable prognosis [2].

Compared to conventional cancer treatments, targeted therapies are expected to be more effective due to their enhanced precision which can negate detrimental side effects on normal cells. Until recently, only two targeted therapies have been approved for treating recurrent OC: the anti-angiogenic immunotherapy Bevacizumab (antibody targeting vascular endothelial growth factor (VEGF)) and PARPi (poly-ADP–ribose polymerase) inhibitors [2]. Whilst these agents have improved the prognosis of epithelial OC, issues around acquired Bevacizumab and PARPi resistance have emerged in clinic [3,4]. VEGF isoform diversity has been described as potential mechanism for OC cells to evade bevacizumab action [3]. In addition, significantly elevated levels of hypoxic-induced proteins involved in the formation of new blood vessels have been identified in patients with EOC who did not respond to bevacizumab treatment [4]. Mechanisms of resistance to PARPi therapy include mutations in PARP1 that diminish the binding of PARPi or allow PARP1 to maintain endogenous functions, post-translational modifications of PARP1 which result in reduced binding by PARPi, restoration of at least partial Homologous Recombination capabilities, stabilization of replication forks, and upregulation of drug efflux pumps [5].

Additionally, platinum-resistant epithelial OC outcomes are extremely poor, with an average of 12 months median survival [6]. To expand the efficacy of these agents and improve OC outcomes, the need for novel therapeutic strategies and combination therapies are urgent. One of the novel therapeutic strategies against ovarian and other forms of cancer that show promising potential for targeted therapy is the use of antibody drug conjugates (ADC). ADCs are designed to target malignant cells without harming healthy cells, while reducing adverse effects and risk of recurrence to accelerate recovery time [7]. They are typically composed of monoclonal antibody (mAb), linker, and cytotoxic payload. The mAb enables the circulation of ADC within the bloodstream prior to binding to the tumour-specific surface antigen. Following binding, the receptor–ADC complex usually internalizes (although some ADCs may be cleaved extracellularly, causing bystander effect) through the endocytosis pathway, leading to the death of cancer cells following proteolytic degradation of the antibody and release of the payload into the intra-cellular environment [8]. Major advantages of ADCs include specific binding to the target antigen, thus maximizing safety and efficacy. A total of 13 ADCs have so far received regulatory approval by the FDA for use in the USA, while four ADCs have been approved by the European Medicines Agency (EMA) [9]. Last year, mirvetuximab soravtansine, an ADC-targeting folate receptor was approved for the treatment of platinum-resistant ovarian cancer [10]. Currently, there are several other ADC candidates developed against ovarian cancer at different stages of clinical trials, including ABBV-428, a mesothelin-CD40 bispecific [11], PF-06650808, an anti-Notch3 antibody-drug conjugate [12], and many other promising ADCs under development [13].

This biomarker-driven therapeutic strategy can be applied to treat ovarian tumours, which are characterised by differentially expressing tumour-antigens [14]. However, there is an increasing need to enhance selection of early-stage molecules to mitigate late-stage failures. For instance, the strength and selectivity of antigen binding, ADC internalization efficiency, and tumour penetration dictates the effectiveness of ADC therapy and are features that must be validated at the earliest stages of ADC development. The elucidation of these characteristics falls to the optimization of biochemical and analytical techniques used to study structural and functional characteristics such as affinity, kinetics, potency, solubility, stability, immunogenicity, and pharmacokinetics, in addition to well-established cell-based assays to study off-target effects and toxicity.

MAb–antigen affinity and stability properties of ADCs have been studied using surface plasmon resonance (SPR) [15]. SPR is a well-established optical detection technique utilised for the measurement of strength and rate of biomolecular interactions [16,17]. The label-free detection negates the need for radioisotope tags or fluorescent reporter molecules which can be expensive, time-consuming, and could influence the molecular interactions. The real-time measurement capability allows for the calculation of association (on-rate, _ka_) and dissociation (off-rate, k_d_) rate constants and equilibrium dissociation constant (K_D_). Specifically, the association rate measures how fast association of the ligand and analyte occurs, while the dissociation rate measures the dissociation. The K_D_ is the ratio between the dissociation and association rate between the antibody and its antigen, which is inversely correlated to the binding affinity. In addition, the microfluidic system of SPR platforms enables the use of small amounts (microlitres) of reagents for assay development and is advantageous when compared with other traditional endpoint-only equilibrium measurement immunoassays for measuring antibody–antigen interaction such as enzyme-linked immunosorbent assay (ELISA). The affinity characteristics of mAbs and ADCs have been investigated using SPR previously [16,18,19]. In therapeutic antibody development, SPR is part of a range of analytical methods also used to study drug-target binding interactions, stability, and binding to Fc receptors [19].

An important aspect of ADC development is the identification of an appropriate target molecule that is specifically overexpressed within cancerous tissue compared to normal tissue [18]. Therefore, in this current study, we demonstrate a complete pipeline for the selection of ADC targets. We highlight a novel bioinformatic strategy for identifying novel potential ovarian cancer targets. Subsequently, we demonstrate a rigorous target validation pipeline comprising target cellular localization, expression levels in normal vs. cancer state, expression levels in 2D and 3D models, and the development of a simple and refined methodology to analyse antibody–antigen binding via SPR and cellular antibody internalization to identify ideal targets for ADC development. Specifically, we identify and validate five targets overexpressed in ovarian cancer, of which three demonstrate significant antibody internalization capacity, making them promising candidates for future ADC development.

## 2. Materials and Methods

### 2.1. Online Tool/Public Data Source

The ADC target database is a MySQL database with a schema optimised for queries which efficiently select and rank potential ADC target proteins in various cancers. Ideally, potential targets should (1) be highly expressed in the cancer tissue, (2) have low expression in healthy tissues, and (3) be localized to the cellular membrane.

Datasets for the ADC database for protein characteristics, including cellular localization and protein expression in healthy and cancer tissues, were obtained from online databases: (1) UniProt https://www.UniProt.orgl (accessed on 12 November 2018) [20], (2) ENSEMBL https://www.ensembl.org/index.html (accessed on 12 November 2018) [21], and (3) the human protein atlas (HPA) https://www.proteinatlas.org/ (accessed on 12 November 2018) [22,23]). UniProt and ENSEMBL databases were queried programmatically using the REST API via custom Python 3 scripts. For each known protein in the human proteome, UniProt was queried for (1) Protein ID, (2) protein sequence, (3) ENSEMBL Gene ID (for gene encoding the protein), (4) sub-cellular localization, and (5) whether the protein is transmembrane. ENSEMBL was queried for gene symbols, based on the ENSEMBL Gene IDs obtained from UniProt.

Data relating to protein expression in healthy tissue and corresponding cancerous tissues was obtained from the human protein atlas (HPA). HPA was queried programmatically by iterating through each ENSEMBL ID obtained from UniProt via a custom Python 3 script. For each ENSEMBL ID, an XML file containing all HPA annotations for the corresponding gene was read into memory. Each XML file was parsed for (1) IHC protein expression results for all healthy tissues and (2) IHC protein expression results for all cancerous tissues. To obtain predictions of the number of transmembrane regions, protein sequences downloaded from UniProt were concatenated into FASTA format and uploaded to the TopCons web-server https://topcons.cbr.su.se/ (accessed on 28 January 2019) [24]. TopCons provided transmembrane prediction results from five separate transmembrane prediction algorithms, with a sixth prediction performed using the TopCons consensus procedure. All data were stored as text files or Python pickle files. The MySQL database tables were generated, and the data were written to them using Python 3 scripts by utilising the MySQL connector library. Following creation, the database was indexed to increase search performance.

Finally, AbDesigner, an online tool used for identifying optimal immunizing peptides for antibody production using a peptide-based strategy [25] https://esbl.nhlbi.nih.gov/AbDesigner/ (accessed on 14 March 2019), was utilized for the identification and selection of optimal peptide immunogen sequence for antibody selection against specific regions of target proteins.

Images related to immunohistochemistry protein expression on ovarian and cancer tissue were taken from the HPA [22,23] (representative IHC Images links: https://www.proteinatlas.org/ENSG00000082556-OPRK1/pathology/ovarian+cancer#ihc, GABRB1 images taken from HPA October 2019 version, https://www.proteinatlas.org/ENSG00000070018-LRP6/pathology/ovarian+cancer#ihc, https://www.proteinatlas.org/ENSG00000120324-PCDHB10/pathology/ovarian+cancer#ihc, https://www.proteinatlas.org/ENSG00000113248-PCDHB15/pathology/ovarian+cancer#ihc) (accessed on 14 October 2019).

### 2.2. Pathway Analysis

Overrepresentation pathway analysis was performed using the WebGestalt 2019 web (Houston, TX, USA) interface [26]. Ensembl gene IDs for genes encoding the 100 highest-ranked target proteins were uploaded to identify enriched pathways in GO Biological Process, KEGG, Panther, and REACTOME functional databases [27,28,29,30]. The ‘genome protein-coding’ reference set was used for the reference gene list. Pathway analysis results were plotted using the R ggplot2 package [31].

### 2.3. Cell Culture

SKOV-3 and OVCAR-3 wild-type (WT) cells, as well as HOSEpiC cells, were purchased from the American Type Culture Collection (ATCC, Manassas, VA, USA), while cisplatin-resistant SKOV-3 and OVCAR-3 cell lines were provided by Axis Bioservices (Coleraine, UK). Both sensitive and resistant cells were cultured in McCoy’s media (Cat 26600-023, Gibco, Leicestershire, UK), containing 10% Fetal Bovine Serum (FBS) and 5% penicillin/streptomycin, and RPMI 1640 media, containing 20% FBS, 1% penicillin/streptomycin, and 0.01 mg/mL bovine insulin. HOSEpiC, UACC-1598, and UWB-1289 cell lines were cultured in Dulbecco’s Modified Eagle Medium: Nutrient Mix F-12 (DMEM/F12), supplemented with 10% FBS and 1% penicillin/streptomycin. COV644 was cultured in DMEM (Cat 10-017-CVR, Corning, NY, USA) supplemented in 15% FBS and 1% penicillin/streptomycin. TOV112D and TOV21G were both cultured in Medium 199 (Cat M4530, Sigma, Welwyn Garden City, UK) and supplemented with MCDB105 (Cat M6395, Sigma, Welwyn Garden City, UK) dissolved in sterile filtered water, 15% FBS, and 1% penicillin/streptomycin. A2780 WT and cisplatin-resistant cell lines were cultured in RPMI 1640 media containing 10% FBS and 1% penicillin/streptomycin. During alternate cell passages, media for cisplatin-resistant cell lines was additionally supplemented with cisplatin at final concentrations of 3 μM for SKOV-3cis, 1.5 μM for OVCAR-3cis, and 1 μM for A2780cis cells. Cells were subsequently maintained at 37 °C in a 5% CO_2_ humidified incubator.

Three dimensional spheroids derived from SKOV-3 WT cell lines were cultured by the liquid overlay technique [18]. SKOV-3 cells grown as a two-dimensional monolayers were resuspended with trypsin, and 5 × 10^3^ cells were seeded in 200 μL of appropriate culture medium and cultured in Ultra-Low Attachment Multiple Well Plate (Cat 7007, Corning, NY, USA) to obtain a single spheroid per microwell. Spheroids were subsequently harvested 72 h post-culture for immunofluorescence assay.

Brightfield images were acquired from Zeiss PrimoVert light microscope equipped with Axiocam ERc 5 s camera (Carl Zeiss Microscopy GmbH, Jena, Germany). All cell lines were verified with the MycoAlert mycoplasma detection kit (Lonza, Castleford, UK) and experiments involving these cell lines were conducted between passages 4 and 20 following thawing.

### 2.4. Western Blot

Protein quantification was performed (Bradford assay) and equal amounts (20 μg) were resolved by SDS-PAGE, transferred to PVDF membranes, and blocked overnight with 5% BSA in 0.1% Tween-20-TBS (TTBS). For recombinant protein detection, 15 ng was loaded, due to the high sensitivity during antibody detection. Subsequently, membranes were incubated at 4 °C with the appropriate primary antibodies diluted 1/1000 in 5% BSA-TTBS buffer. Blots were further incubated for 1 h with IgG horseradish peroxidase secondary antibody diluted 1/2000 in 5% BSA-TTBS buffer. Between incubation steps, membranes were washed 3 × 10 min with TTBS. Blots were analysed for GAPDH levels (GAPDH rabbit polyclonal antibody FL-335, Santa Cruz Biotechnology, Dallas, TX, USA) to normalise protein loading in each well. Immunoreactive bands were visualized using a ChemiDoc System Bio-Rad Imager (Bio-Rad laboratories; Hertfordshire, UK).

### 2.5. Gene Expression Analysis

Gene expression analysis was carried out by interrogating the GEPIA2 public portal database, which references TCGA datasets and GTEx datasets. Expression levels of the OPRK1, GABRB1, LRP6, PCHDB10 and PCDHB15 were addressed in biopsies of ovarian cancer vs. the matched healthy tissue, with a significance threshold set at *p* < 0.05, according to the tool: Expression analysis/Expression DIY/Box plot, in GEPIA2 portal. qRT-PCR experiments were also performed to identify expression levels of the targets using healthy and ovarian cancer cell lines, as described before [18]. Briefly, RNA was extracted from cell lines using the RNeasy^®^ Mini Kit (Qiagen, Hilden, Germany) according to the manufacturer protocol, from which cDNA was generated using the High-Capacity cDNA Reverse Transcriptase Kit (Applied Biosystems by Thermo Fisher Scientific, Loughborough, UK). Reverse transcription reactions were performed using the T100™ Thermal Cycler (Bio-Rad laboratories, Hertfordshire, UK). Samples were analysed by qPCR in triplicate using the iTaq™ Universal SYBR^®^ Green Supermix (Bio-Rad laboratories, Hertfordshire, UK), run on the CFX96 Real-Time PCR Detection System (Bio-Rad laboratories, Hertfordshire, UK), using specific primers for RPS18 (forward: 5′-ATTGCCGACAGGATGCAGAA-3′, reverse: 5′-GCTGATCCACATCTGCTGGAA-3′), OPRK1 (forward: 5′-CTGCTGTCGTCATCTGTTGG-3′, reverse: 5′-GCACTCAATGACATCGACGT-3′), GABRB1 (forward: 5′-AATCCCACTGAACCTCACCC-3′, reverse: 5′-TGACCCCATGCACAAATGAT-3′), LPR6 (forward: 5′-ATGCAAACAGACGGGACTTG-3′, reverse: 5′-AAACACAAAGTCCACCGCAG-3′), PCDHB10 (forward: 5′- AACTACACGATCAGCCCCAA-3′, reverse: 5′-TCCAGTGCTTTGTCCAACAC-3′), and PCDHB15 (forward: 5′-CCATCACAGACTTGGGGACT-3′, reverse: 5′-CGAACAGGGTGTAGGAGGTT-3′). Serial dilutions of cDNA were used to plot a calibration curve, and gene expression was quantified by plotting threshold cycle values. Expression levels were normalised to values obtained for the reference gene RPS18. Relative expression was expressed as the average ± standard deviation.

### 2.6. Healthy Tissue Lysates

The following healthy human tissue lysates were purchased; human brain whole-tissue lysate (NB820-59177, Novusbio, Abingdon, UK), human breast whole-tissue lysate (NB820-59203, Novusbio), human kidney whole-tissue lysate (NB820-59231, Novusbio), human lung whole-tissue lysate (NB820-59239, Novusbio), human liver whole-tissue lysate (NB820-59232, Novusbio), human pancreas whole-tissue lysate (NB820-59244, Novusbio), human spleen whole-tissue lysate (NB820-59259, Novusbio), human ovary whole-tissue lysate (NB820-59243, Novusbio), human uterus whole-tissue lysate (NB820-59274, Novusbio). Upon delivery, lysates were aliquoted and stored at −80 °C. When needed, protein lysates were used to perform western blot analysis, as described above.

### 2.7. Antibodies and Recombinant Proteins

Primary antibodies used for western blot, SPR off-rate screening, high content image analysis using an INCell Analyzer 6000 (Molecular Devices, San Jose, CA, USA), and confocal microscopy (LSM 510, Carl Zeiss Microscopy GmbH, Jena, Germany) are recapitulated in Supplementary Table S3. The corresponding recombinant proteins to each primary antibody were utilized as follows: PrEST LRP6 [180 kDa (Sigma)], PrEST PCDHB10 [87 kDa (Sigma)], PrEST PCDHB15 [86 kDa (Sigma)], rHuman OPKR1 [42 kDa (Abcam, Cambridge, UK)], rHuman GABRB1 [54 kDa (Abcam)].

### 2.8. Off-Rate Screening

Biacore™ T200 system (Cytiva, Uppsala, Sweden) was used to perform SPR analysis with Series S Sensor Chip CM5 (Cytiva, 29149603) and HBS-EP+ (0.01 M Hepes, 0.15 M NaCl, 0.003 M ethylenediaminetetraacetic acid, and 0.05% Surfactant P20, pH 7.4) (Cytiva, BR100669) as sample and running buffer. The analysis temperature at the sensor chip surface and within the compartment was set to 25 °C. Firstly, pH scouting with 10 mM sodium acetate buffer 4.0 (Cytiva, BR-1003-49), 10 mM sodium acetate buffer 4.5 (Cytiva, BR-1003-50), 10 mM sodium acetate buffer 5.0 (Cytiva, BR-1003-51), and 10 mM sodium acetate buffer 5.5 (Cytiva, BR-1003-52) was performed to identify the optimal pH required for amine coupling of ligands to Series S Sensor Chip CM5. This was followed by diluting the ligand (primary antibody) with the appropriate acetate buffer to at least 10 μg/mL. Amine coupling of the ligand to Series S Sensor Chip CM5 was then performed using Amine Coupling Kit (Cytiva, BR100050) containing 1-Etyhyl-3-(3-dimethylaminopropyl)-carbodiimide hydrochloride (EDC), *N*-Hydroxysuccinimide (NHS), and 1.0 M ethanolamine-HCL, at pH 8.5, in accordance with the manufacturer’s instructions. For each experimental run, the ligand was immobilized in flow cells 2 or 4, while flow cells 1 or 3 were left unmodified. Flow cells 2 and 4 were used as active flow cells while flow cells 1 and 3 were used as reference flow cells. Ligand was injected at a flow rate of 10 μL/min and a contact time of 300 s. Analytes (recombinant proteins) were injected for 120 s with a flow rate of 30 μL/min and dissociation time of 600 s, run in order of increasing concentration over the reference and active flow cells using a multi-cycle kinetic approach with five–seven concentration range. Following each binding cycle, the surface was regenerated with a 30 s injection of 10 mM Glycine-HCl, pH 2.5 (Cytiva, BR-1003-56) at 30 μL/min, which removed the bound antigen. Blank cycles (buffer-only injection followed by regeneration step) were also performed. Data were double-referenced by first subtracting responses from the reference flow cell and then subtracting the blank cycles. Data were then fitted to a 1:1 dissociation model using Biacore^TM^ Insight Evaluation Software version 3.2.1 (Cytiva, Uppsala, Sweden).

### 2.9. Immunofluorescence Assay

The immunofluorescence assay was performed following standard procedures when cells were cultured as 2D monolayers. Briefly, cells (1.5 × 10^4^) suspended in 100 μL of phosphate-buffered saline (PBS) were plated on 8-well chamber slides (155411, Thermo Fisher Scientific) following cytospin at 800 rpm for 3 min. Once 80–90% confluency was achieved, the cells were fixed in 4% paraformaldehyde, blocked with 3% BSA, and washed in 3% BSA and 0.1% phosphate-buffered saline (PBS)–Tween-20 [18]. The cells were incubated overnight at 4 °C with the appropriate primary antibody followed by secondary Alexa Flour 594 cross-absorbed labelled antibody (Life Technologies, Loughborough, UK, A11012) at 1:500 dilution and DAPI (Invitrogen, Loughborough, UK, P36962) at 1:2000 dilution.

The following protocol, according to Weiswald et al. [32], was used to assess immunofluorescent signal in 3D spheroids. Approximately 50 spheroids in suspension were fixed and permeabilized for 3 h at 4 °C containing 4% PFA (Euromedex, Mundolsheim, France) and 1% Triton X-100 (T8787-250 mL, Sigma-Aldrich, St. Louis, MO, USA) and washed in PBS (2 × 10 min). The spheroids were subsequently dehydrated in an ascending order in methanol at 4 °C in PBS (25%, 50%, 75%, 95%, 15 min each and 100% for 30 min). Rehydration was performed in the reverse descending order and washed in PBS for 10 min. Following blocking in PBST (0.1% Triton X-100 in PBS) containing 3% Bovine Serum Albumin (Cat P06-1391050 PAN Biotech, Issy-les-Moulineaux, France) overnight at 4 °C with washing in PBST for 10 min, spheroids were incubated with primary antibodies in PBST on a low-speed rotator at 4 °C for 48–72 h and rinsed in PBST for 10 min. Spheroids were then incubated in appropriate Alexa Fluor conjugated secondary antibodies, diluted in PBST overnight at 4 °C. The spheroids were then transferred to 8-well chamber slides and incubated in DAPI in 1:2000 dilution in PBS at room temperature for 30 min prior to visualization.

Images were acquired from a Zeiss LSM 710 confocal microscope (Carl Zeiss Microscopy, Jena, Germany) and analysed with the Zen 2012 (blue/black edition) image analysis software (Carl Zeiss Microscopy, Jena, Germany).

### 2.10. pHAb Amine Antibody Internalization Assay

Internalization of the appropriate antibodies was performed according to Healey et al. [18]. Antibody conjugation to the pHAb Amine Reactive Dye and DAR calculations were performed according to the manufacturer’s recommendations (Cat. No. G983, Promega, Southampton, UK). The pHAb Amine is a pH-sensitive dye that is non-fluorescent at neutral pH (extracellular, culture medium) and fluorescent at acidic pH (lysosomes, intracellular, endosomes). OVCAR3 WT and OVCAR3 cisplatin-resistant cell lines were used to study internalization of pHAb conjugated antibodies. Firstly, target antibodies were conjugated with the pHAb dye according to manufacturer’s guidelines. The cells were then seeded (1 × 10^4^ cells/mL) in a 96-well plate (215006, Porvair, New Milton, UK) in 200 μL of appropriate media and cultured for 24 h in a humidified, 5% CO_2_ incubator at 37  °C. Once 90–95% confluency was attained, cells were washed in PBS and treated with pHAb conjugated antibodies at 10 μg/mL, then incubated on ice for 30 min. The cells were then transferred to the incubator at 37 °C for 240 min. To achieve higher sensitivity, media were replaced with PBS before image acquisition. Control wells were treated with only PBS, and DAPI and fluorescence signal was used as background from the cells and subtracted from positive wells. Images were acquired on an INCell Analyzer 6000 (Cytiva, Uppsala, Sweden).

### 2.11. Cell Viability

A positive control, Staurosporine (J62837, Alfa Aesar, Ward Hill, MA, USA), was dissolved in DMSO (dimethyl sulfoxide) to make a 10 mM stock solution. Cells were seeded in white-walled 96-well plates (Porvair Sciences, Wrexham, UK) at densities of 2500 cells/well. Then, 24 h following seeding, the medium was removed and replaced with a medium containing 1 μM of the drug or the 5 target antibodies. Subsequently, the treatment medium was supplemented with RealTime-Glo MT Cell Viability Assay (Promega, Madison, WI, USA) reagents, according to manufacturer-recommended concentrations (1:1000). Treated samples were kept in a cell culture incubator, and luminescence per well was measured every 24 h in a microplate photometer at 37 °C.

### 2.12. Statistics

Data were analysed for normality using the Kolmogorov Smirnov test. Normally distributed data were analysed using the parametric Student’s *t*-test (2-tailed) method (Minitab 16), and *p*-value ≤ 0.05 was considered statistically significant. The Chi^2^% value was calculated by dividing the Chi^2^ from the 1:1 dissociation model by the R_max_ from the 1:1 standard model and multiplying by 100 to convert to percentage. Values below 5% are of high significance. The Chi^2^, Chi^2^%, and k_d_ values were all calculated using Biacore™ Insight Evaluation Software, version 3.2.1 (Cytiva, Uppsala, Sweden).

## 3. Results

### 3.1. Identification of Candidate Targets via ADC Database

#### 3.1.1. In Silico Database Development

**Figure 1:**
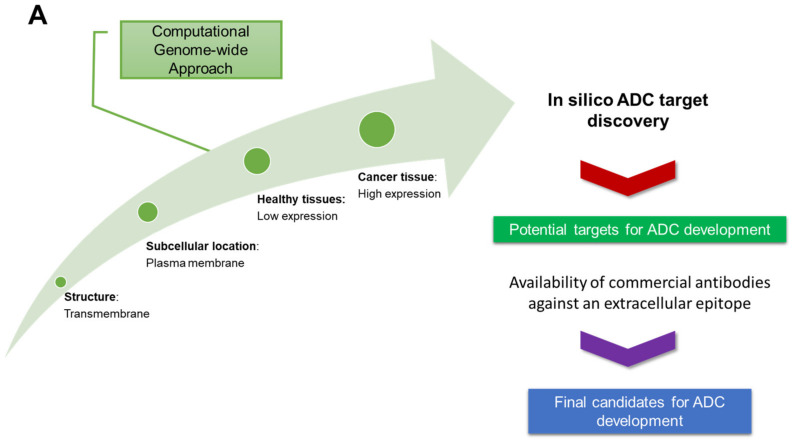

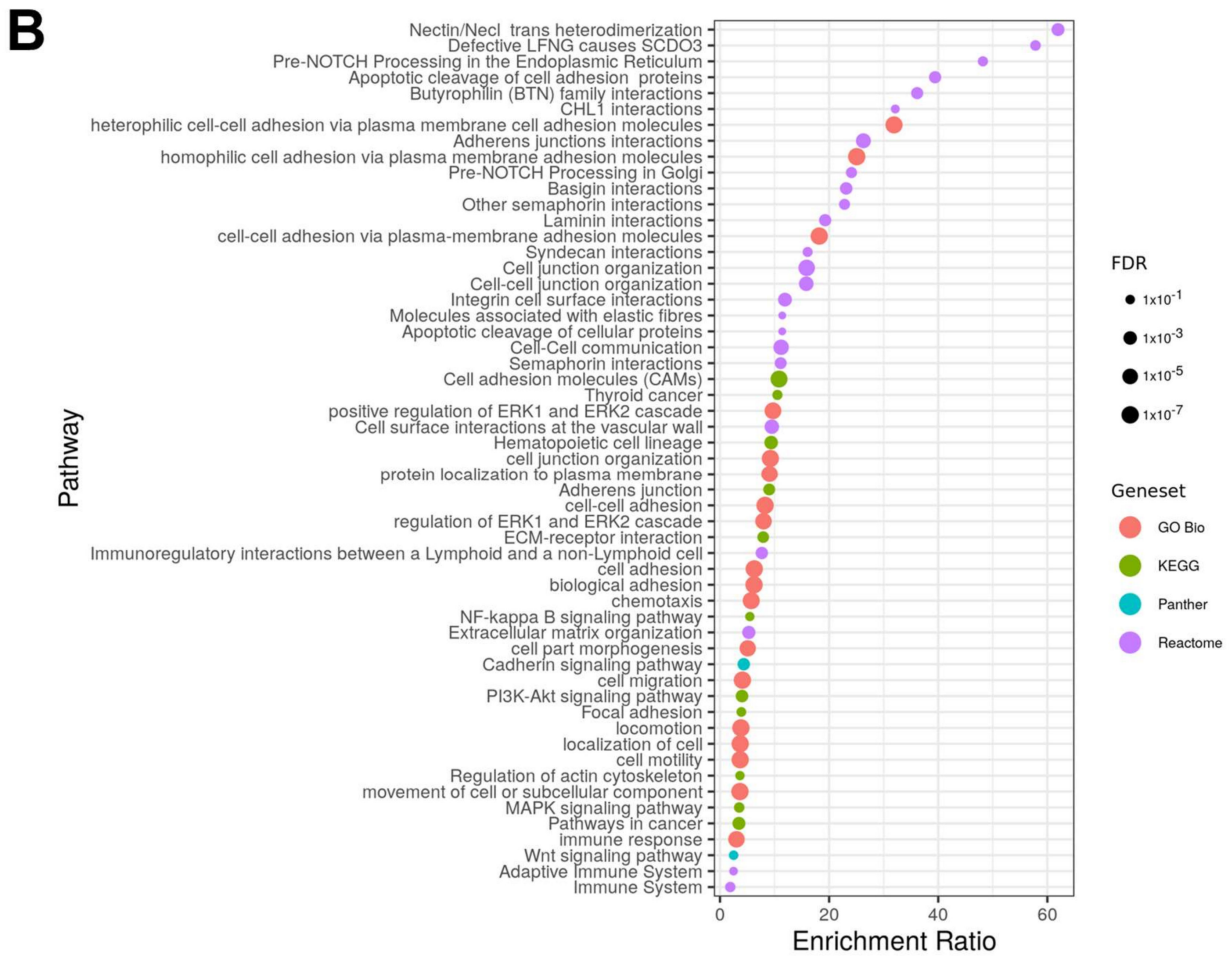
Creation of MySqL Database. (**A**) Schematic representation of the steps applied in the creation of MySqL database, ADC Target Vault, providing a list of ADC ovarian cancer target candidates. (**B**) Enriched pathways for gene-encoding proteins associated with the top 100 targets identified using the ADC database. ORA results indicating significantly enriched pathways (FDR < 0.1) from GO Biological Process, KEGG, Panther, and REACTOME functional databases for top 100 highest-ranked targets.

To identify potential targets for ADCs in OC, an in silico approach was implemented, taking advantage of the extensive range of bioinformatics databases and tools available online (Figure 1). The following properties were taking into consideration for selection of effective ADC target proteins: (i) transmembrane proteins to enable antibody binding at the extracellular domain, (ii) overexpression in target cancer cells, and (iii) low expression in healthy cells.

We created a MySQL database, termed ADC Target Vault, with a schema designed to optimize queries relating to the above-mentioned ADC properties (Supplementary Figure S1). Tables were populated with data obtained from online databases and from results obtained from running transmembrane prediction tools on human protein sequences (Supplementary Tables S1 and S2). Briefly, human protein sequences were obtained from UniProt. And Protein/Gene mapping data were obtained from ENSEMBL. Data relating to protein expression levels for both healthy tissue and OC tissue (from IHC experiments) were obtained from the human protein atlas (HPA). Finally, transmembrane prediction results (predicting the number of transmembrane domains that potential targets contain) were obtained from six transmembrane prediction algorithms by uploading all known proteins found in the human proteome to TopCons webserver. For full details of database initialization and population, see Methods section. Since the ADC target database contains expression data for all cancer types annotated in HPA, it may be queried for suitable ADC targets for any of these cancers. In the present study, we illustrate the pipeline from bioinformatic analysis to in vitro target validation and antibody selection for ovarian cancer targets.

To generate a list of potential ovarian cancer biomarkers suitable for ADC targeting, the database was queried for proteins with >50% of patients exhibiting high or medium expression in OC. Potential targets were ranked by (1) Lowest number of healthy tissues with high and medium expression of combined proteins, (2) Lowest number of healthy tissues with high expression of protein, (3) Lowest number of healthy tissues with medium expression of protein, (4) Highest percentage of patients with high and/or medium expression of protein in cancerous tissue. Query results were written to csv format with a query time of approximately 20 s. Querying for ovarian cancer targets returned 1207 possible targets, with the top 20 targets further investigated and validated as suitable ADC targets. Results were filtered to only include proteins with a single transmembrane spanning domain, as predicted by at least five out of six transmembrane prediction algorithms, including TOPCONS [24], Philius [33], PolyPhobius [34], SPOCTOPUS [35], OCTOPUS [36] and SCAMPI [37]. Overrepresentation analysis was performed to identify enriched pathways for the genes encoding the top 100 target proteins in GO Biological Process, KEGG, Panther, and REACTOME functional databases (FDR < 0.1). Enriched pathways are shown in Figure 1B. The three pathways with highest enrichment from the GO Biological Process database were cell–cell adhesion via plasma membrane adhesion molecules pathways. Interestingly, ERK1 and ERK2 cascade pathways are also enriched. ERK dysregulation is associated with tumorigenesis [38].

For KEGG, the three pathways with higher enrichment are ‘Cell Adhesion Molecules’, ‘Thyroid cancer’, and ‘Hemetopoietic cell lineage’. From Panther, the ‘Cadherin signaling pathway’ and ‘Wnt signaling pathway’ are enriched. For REACTOME, the three pathways with highest enrichment were Nectin/NEcl trans heterodimerization, Defective LFNG caused SCDO3, and Pre-NOTCH Processing in the Endoplasmic Reticulum.

#### 3.1.2. Target Expression in Healthy and Ovarian Cancer Tissues

**Figure 2:**
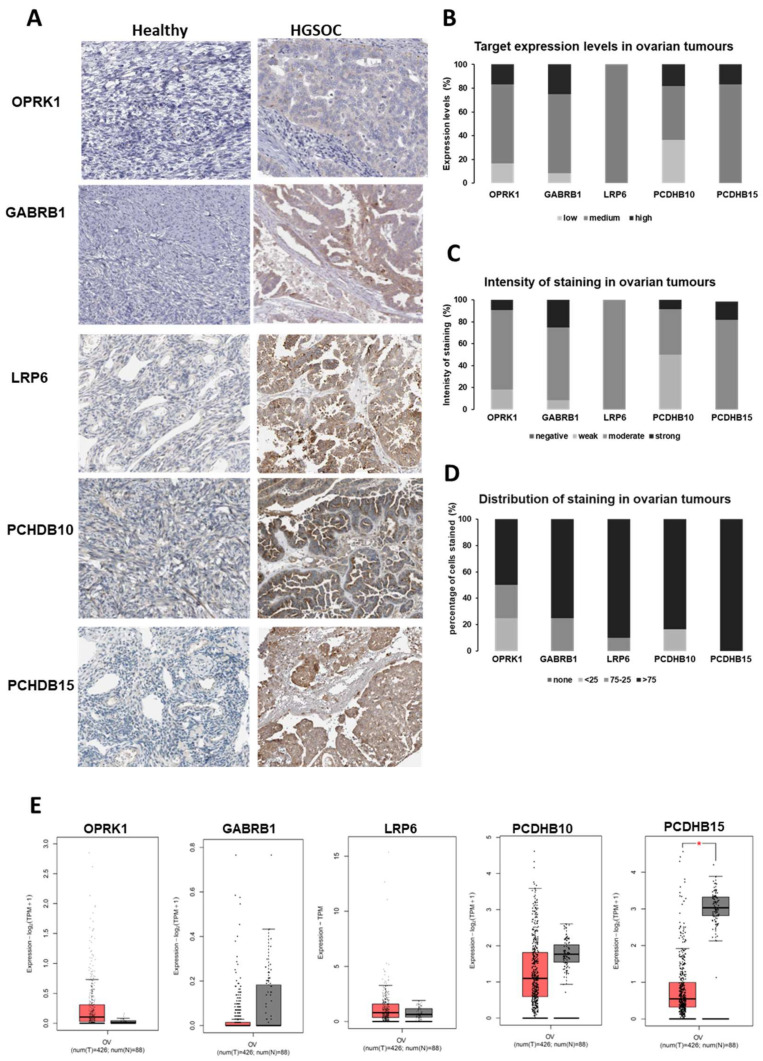
Target expression characteristics in ovarian cancer vs. healthy tissue. (**A**) Expression data of the 5 targets in ovarian cancer tumours obtained from HPA (**B**) Target expression, (**C**) Staining intensity, and (**D**) Staining distribution. (**E**) Gene expression levels between ovarian cancer (red, *n* = 426) and healthy ovary tissue (grey, *n* = 88) were obtained from GEPIA2. * *p*-value < 0.05.

Data from These Top 20 Targets Was Obtained from HPA (Figure 2A). The Intensity and Distribution of Staining within the Tumour Sections Was Also Scored Using the HPA Published Slides (Figure 2B–D).

Data obtained from these 22 ovarian carcinoma slides revealed a medium to high level of expression for all the top 20 targets selected and a strong to moderate intensity of staining in all patient samples (Figure 2A,B). Moreover, the distribution of staining of these biomarkers within the tissues showed that PCDHB15 was homogenously expressed within the sections, with more than 75% of cells expressing the target in all patient samples (Figure 2C). GABRB1, PCDHB10, and LRP6 also showed that 80% to 90% of the patients exhibited expression of the biomarker in more than 75% of tumour cells. Only 65% of patients exhibited a high distribution of staining for OPKR1 in the tissue slides (Figure 2C). Next, we selected OPKR1, GABRB1, LRP6, PCDHB10, and PCDHB15 as targets of interest based on antibody availability for validation. Because our database does not filter targets according to their mRNA levels, we also interrogated the gene expression levels of these targets using the public GEPIA2 database (Figure 2E). No statistically significant differences between cancer and healthy ovarian tissue were observed for the targets (except for PCDHB15), nor was a correlation between gene expression levels and protein levels for these targets.

**Figure 3:**
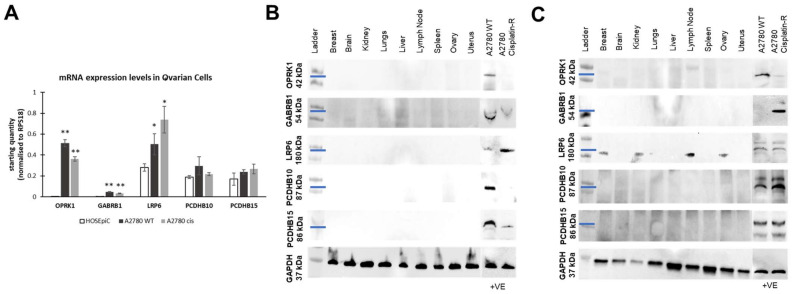
Target expression in healthy versus ovarian cancer tissue and cell lines. (**A**) Analysis of mRNA levels for all the targets was performed by qRT-PCR. (**B**,**C**) Protein expression analysis of the targets was performed in healthy tissue lysates, ovarian cancer A2780 WT, A2780 cisplatin-resistant cell lines (positive control), and evaluated via western blotting by utilizing primary antibodies targeting the (**B**) *N*-terminus and (**C**) C-terminus of the targets. Results were normalized using GAPDH as a housekeeping protein. The blue line reflects the approximate MW the target using the MW marker as reference. * *p*-value < 0.05, ** *p*-value < 0.01.

We also analysed the mRNA levels of the targets in healthy (HOSEpiC) and ovarian cancer cell lines by qRT-PCR (Figure 3A). Gene expression levels for OPRK1 and GABRB1 were extremely low in healthy cells and upregulated in ovarian cancer cisplatin-sensitive and -resistant cells. LRP6 mRNA levels were significantly upregulated in ovarian cancer cells compared to healthy ones (Figure 3A). No significant differences were observed for PCDHB10 or PCDHB15 mRNA levels between healthy and OC cells.

One of the major advantages of ADC therapies is that they are designed to target antigens overexpressed in cancer tissues and minimally expressed or absent in healthy tissues to minimize systemic toxicities [9]. Commercially available protein lysates from healthy tissue samples corresponding to vital human organs were analysed for the respective target expression by western blotting. We utilized two different sets of antibodies targeting different epitopes of each target (Supplementary Table S3); one targeting the *N*-terminus (extracellular domain) and another targeting the C-terminus (intracellular domain) were used to evaluate the expression of the different isoforms of the targets and confirm the homogeneity of the targets. We reveal that there was no expression of targets in all the healthy tissue samples using either the *N*-terminus- (Figure 3B) or C-terminus-binding antibodies (Figure 3C), while varying expressions were observed in a range of ovarian cancer cells also used as positive controls.

Overall, these results demonstrated the effectiveness of the in silico selection process, as all selected targets fulfil one of the desirable properties of suitable ADC targets by the homogenous absence of protein expression in healthy tissues.

### 3.2. Off-Rate Screening via SPR Platform as a Strategy to Enhance Antibody Selection

**Figure 4:**
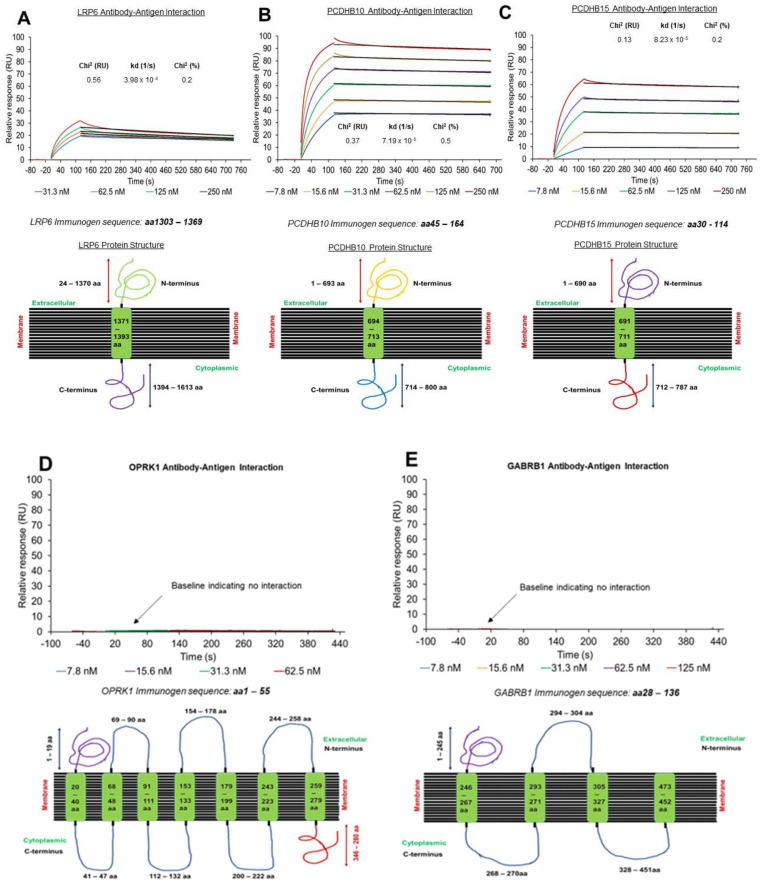
Antibody–antigen dissociation rate (k_off_) profile of targets via SPR analysis. Antibodies were directly attached onto Sensor Chip CM5 via amine coupling. Multi-cycle kinetics experiments were performed. LRP6 antibody (**A**), PCDHB10 antibody (**B**), PCDHB15 antibody (**C**), OPKR1 antibody (**D**), and GABRB1 antibody (**E**) were all exposed to the corresponding recombinant target protein (rLRP6; 31.3 to 250 nM, r PCDHB10; 7.8 to 250 nM rPCDHB15; 7.8 to 250 nM, rOPKR1; 7.8 nM to 62.5 nM and rGABRB1; 7.8 nM to 125 nM). Dissociation rates were determined using one-to-one dissociation model with global Rmax. Representation of the target protein structure highlighting the extracellular, transmembrane, and intracellular regions, as well as immunogen sequence (both antibody and recombinant proteins), are also provided at the bottom of each panel.

Next, we performed a sequence alignment and immunogenicity analysis of the identified proteins with the online software platforms NHLBI-AbDesigner and UniProt to facilitate the development of novel ADCs for gynaecological cancers [16,18]. We utilised commercial antibodies containing immunogen sequences that target the amino acid sequence located within the extracellular region of the target antigen. The location of the amino acid sequence within the target structure was identified using NHLBI-AbDesigner and basic local alignment search with the Basic Local Alignment Search Tool (BLAST) https://blast.ncbi.nlm.nih.gov/Blast.cgi (accessed on 14 March 2019) to analyse sequence alignment of these peptides, in order to verify the location of the conserved region. The analysis enabled the identification of antibodies with the appropriate immunogen sequence that corresponded to the extracellular domain regions of the target antigens as well as within the transmembrane and cytoplasmic regions (Figure 4, bottom panels).

To explore the antibody–antigen interaction, we evaluated the dissociation rate (off-rate, k_off_) for the recombinant (r) target proteins binding to their respective antibody using a Biacore™ T200 instrument. We employed a strategy where antibodies (ligand) were directly immobilized to Sensor Chip CM5 via NHS–EDC chemistry. Experiments were performed by injecting concentration series of recombinant targets proteins rLPR6 (31.3 to 125 nM), rPCDHB10 (7.8 to 250 nM) and rPCDHB15 (7.8 to 250 nM)) over the corresponding immobilized antibodies. The resultant binding profiles for anti-LRP6-rLRP6 (Figure 4A, top panel), anti-PCDHB10-Rpcdhb10 (Figure 4B, top panel), and anti-PCDHB15-rPCDHB15 (Figure 4C, top panel) calculated from the 1:1 dissociation model revealed that binding of the recombinant proteins to their respective antibody was very stable, with off-rates ranging from 4 × 10^−4^ to 8 × 10^−5^ s^−1^. A full kinetics table is also provided (Supplementary Table S4).

Off-rate analyses between anti-OPKR1 and anti-GABRB1 were also performed with rOPKR1 (7.8 to 62.5 nM) and rGABRB1 (7.8 to 125 nM) (Figure 3D,E, top panels), which revealed no binding with the corresponding antibodies, possibly due to comparatively short N’ extracellular domains of these two proteins (Figure 4D,E, bottom panels).

### 3.3. Target Localization and Basal Expression Patterns in Ovarian Cancer Cell Lines

#### 3.3.1. Target Localisation and Basal Expression in Confluent 2D Monolayers

**Figure 5:**
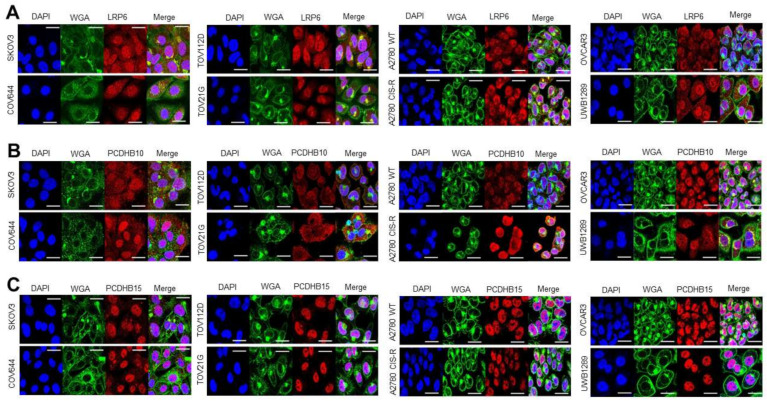

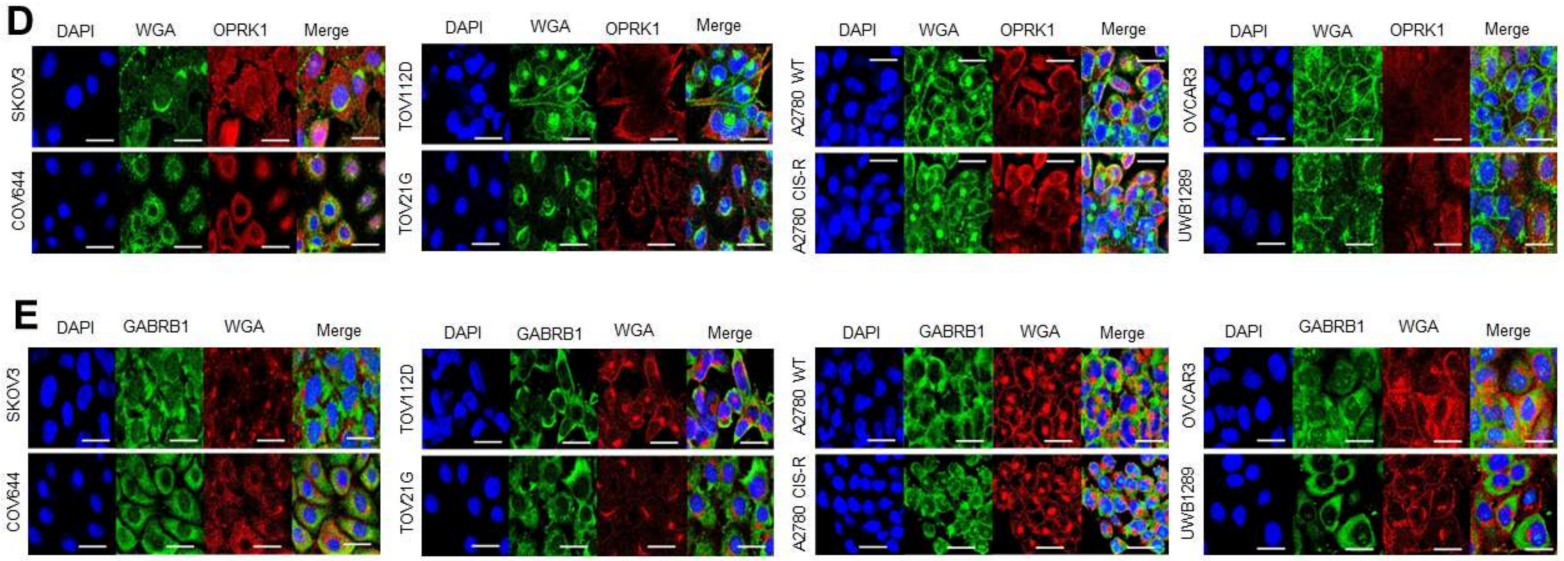
Basal expression and cellular localization of targets in ovarian cancer cell lines. (**A**) Confocal immunofluorescence staining reveals localization of LRP6 (Red), WGA (Green), DAPI (Blue). (**B**) Localization of PCDHB10 (Red), WGA (Green), DAPI (Blue). (**C**) Localization of PCDHB15 (Red), WGA (Green), DAPI (Blue). (**D**) Confocal immunofluorescence staining reveals localization of OPKR1 (Red), WGA (Green), DAPI (Blue). (**E**) Localization of GABRB1 (Green), WGA (Red), DAPI (Blue). Magnification: 40×, Scale bars: 20 μm.

We also sought to confirm the basal expression levels and cellular localization of the target proteins via immunofluorescence assays and confocal microscopy. In ADC development, epitopes on the extracellular region of membrane proteins are usually targeted to facilitate antigen-driven ADC internalization [8]. Using Wheat Germ Agglutin (WGA) staining as a membrane marker, we observed that targets localise at the cell membrane as well as within the cytoplasm in a panel of eight ovarian cancer cell lines (Figure 5). All targets were highly expressed in the eight different ovarian cancer cell lines (Figure 5), but not in the control experiments (Supplementary Figure S2). 

#### 3.3.2. Expression of Target Proteins in 3D Spheroids

**Figure 6:**
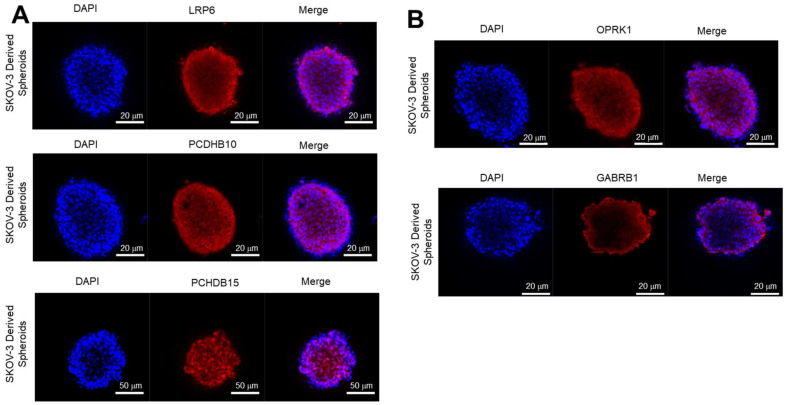
Basal expression of targets in 3D models. Three-dimensional spheroid cultures of SKOV-3 WT were stained with (**A**) anti-LRP6 (top panel), anti-PCDHB10 (middle panel), and anti-PCDHB15 (bottom panel) and (**B**) anti-OPKR1 (top panel), anti-GABRB1 (bottom panel). Cell nuclei were counter stained with DAPI (blue). Stained spheroids were observed under the confocal microscope and merged images are presented.

Target expression was also determined in 3D SKOV3 spheroid models to assess biomarker expression and internalization in conditions that simulate tumour configuration. SKOV-3 spheroids were chosen due to their ability to form compact spheroids without disaggregating over a prolonged period (Supplementary Figure S3). Expression of all the targets was observed in 3D spheroids (Figure 6); however, GABRB1 exhibited the lowest expression which was mainly confined to the outer layers of the spheroid (Figure 6B, bottom panel).

#### 3.3.3. Antibody Penetration in 3D Spheroids

**Figure 7:**
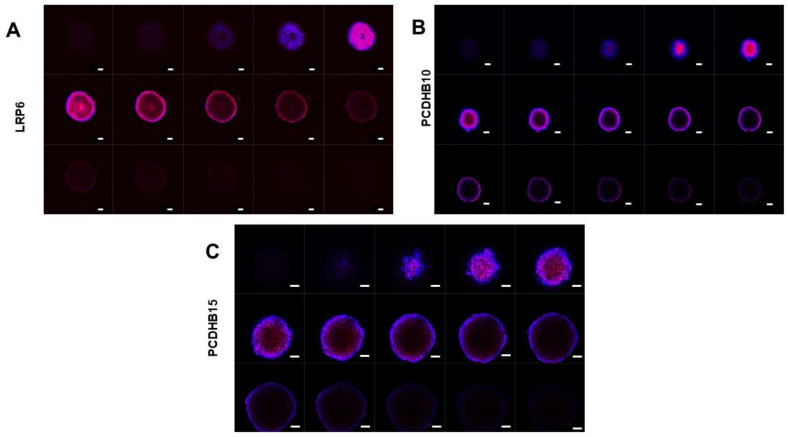
Antibody penetration of spheroids. Z-stack images (15 cross-sectional slices) of SKOV-3-derived spheroids, taken by confocal microscopy, showing antibody penetration of (**A**) anti-LRP6, (**B**) anti-PCDHB10, and (**C**) anti-PCDHB15. Spheroids were stained using immunofluorescence assay with antibodies (Red) and DAPI (Blue) and visualized via confocal microscopy. Magnification: 10×, Scale bars: 50 μm.

Further antibody penetration analysis via confocal z-stacking analysis of the expression and location of LPR6, PCDHB10, and PCDHB15 targets showed that LRP6 was uniformly distributed within the tumour spheroid, whereas PCDHB10 and PCDHB15 localised from the second bilayer of cells to the core of the spheres (Figure 7).

### 3.4. Antibody Internalization in Ovarian Cancer Cells

**Figure 8:**
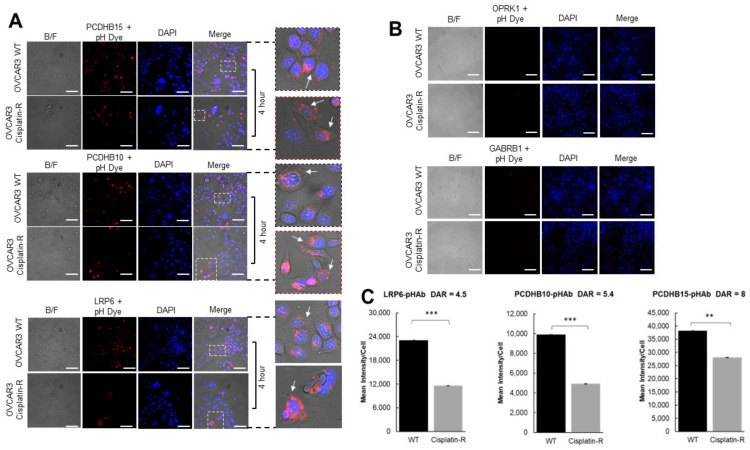
Target antibodies are rapidly internalized and trafficked to the endosomal compartment. (**A**) OVCAR-3 WT and OVCAR3 cisplatin-resistant cell lines were treated with medium containing anti-PCDHB15 (top panel), anti-PCDHB10 (middle panel), and anti-LRP6 (bottom panel) and (**B**) anti-OPKR1 (top panel) and anti-GABRB1 (bottom panel) primary antibody conjugated to a pH-sensitive dye for 4 h. Dotted boxes indicate enlarged cells. Arrows indicate positive (red) internalisation signal (**C**) Histogram showing the quantity of internalization quantified via Cell Profiler software expressed as mean (SD) from 3 independent experiments. Images were acquired on an INCell analyzer 6000 microscope and analysed with ImageJ software. ** *p*-value < 0.01; *** *p*-value < 0.001.

Next, we sought to investigate the rate of antibody–antigen internalization in platinum-sensitive and platinum-resistant OVCAR-3 cells (Figure 8). Here, pHAb-labelled antibodies were used for OPKR1–pHAb (Dye to Antigen Ratio = 3.8), GABRB1–pHAb (Dye to Antigen Ratio = 3.1), LRP6–pHAb (Dye to Antigen Ratio = 4.5), PCDHB10–pHAb (Dye to Antigen Ratio = 5.2), PCDHB15–pHAb (Dye to Antigen Ratio = 8), and internalization monitored in OVCAR3 WT and cisplatin-resistant cells. In this current study, the DAR (Dye to Antibody Ratio) refers to how much pHAb fluorescent dye attaches to an antibody. All recovered conjugated antibodies were within the concentration range for cell-based internalization experiments [35,36]. The internalization assay is based on the pH-sensitive pHAb dye, which has low or no fluorescence at pH > 7, but becomes highly fluorescent at the acidic pH < 7 typically present in early endosomes and lysosomes [18]. Following a 4 h incubation period, internalization was evidenced by the presence of red punctate structures for anti-LRP6, anti-PCDHB10, and anti-PCDHB15, suggesting they had been trafficked to the acidic organelles, including lysosomes and endosomes (Figure 8A). INCell analysis revealed that these antibodies effectively internalized in both OVCAR3 wild-type and cisplatin-resistant cell lines (Figure 8C). OPKR1–pHAb (Figure 7B, top panel) and GABRB1–pHAb (Figure 8B, bottom panel) exhibited no internalization which reflects the lack of target binding revealed via SPR. As a result, further investigation on these two targets was discontinued.

The relationship between antibody–receptor interaction kinetics and receptor-mediated antibody internalization dynamics was also investigated by comparing the antibody–antigen off-rate constant and the quantification of internalization signal for anti-LRP6 and anti-PCDHB10 (Figure 8A,C). PCDHB15 was not included in the analysis as the DAR obtained after conjugation with pH dye was almost double that obtained for LRP6 and PCDHB10 antibodies (Figure 8C). DAR values for anti-LRP6-pHAb (DAR 4.5) and anti-PCDHB10-pHAb (DAR 5.2) were similar, as these were the antibody–antigen on-rate value (Anti-LPR6/recombinant LRP6 k_a_ = 3.68 × 10^5^ 1/Ms and Anti-PCDHB10/recombinant PCDHB10 k_a_ = 3.64 × 10^5^ 1/Ms). Interestingly, quantification data for internalization of these antibodies revealed a 2.4-fold increase in internalization of anti-LRP6-pHAb conjugates compared to anti-PCDHB10-pHAb conjugates. Additionally, the antibody–antigen off-rate constant value for LRP6 was 5.5-fold higher than the one reported for PCDHB10 (Figure 4A,B). These results suggests that antibody/antigen dissociation kinetics could also influence internalization dynamics for antibodies with similar target affinities.

### 3.5. In Vitro Antibody-Mediated Ovarian Cancer Cell Toxicity

**Figure 9:**
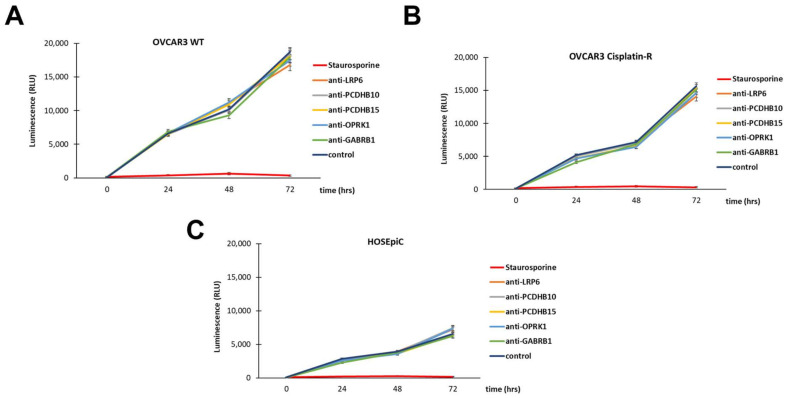
Antibody-mediated ovarian cancer cell toxicity. Cell viability was determined by Realtime-Glo™ MT Cell Viability Assay. OVCAR-3 WT (**A**) and OVCAR-3 cisplatin-resistant (**B**) cells and HOSEpiC healthy ovarian cells (**C**) were treated with Staurosporine, anti-LRP6, anti-PCDHB10, anti-PCDHB15, no treatment, and Staurosporine, anti-OPKR1, anti-GABRB1, no treatment. All cells were treated for 72 h.

Finally, we evaluated whether there was any inherent cytotoxicity associated with the target antibodies following the exposure of HOSEpiC, OVCAR3 WT, and OVCAR3 cisplatin-resistant cell lines. Cells were cultured in the presence of 1 μM target antibodies and staurosporine for 72 h, and the cell viability was determined using the Real-time Glo™ MT Cell Viability Assay. Cells treated with staurosporine showed very low luminescence indicating cell death, while treatment with the target antibodies did not cause any cytotoxic effect on the cells (Figure 9A–C).

## 4. Discussion

**Figure 10:**
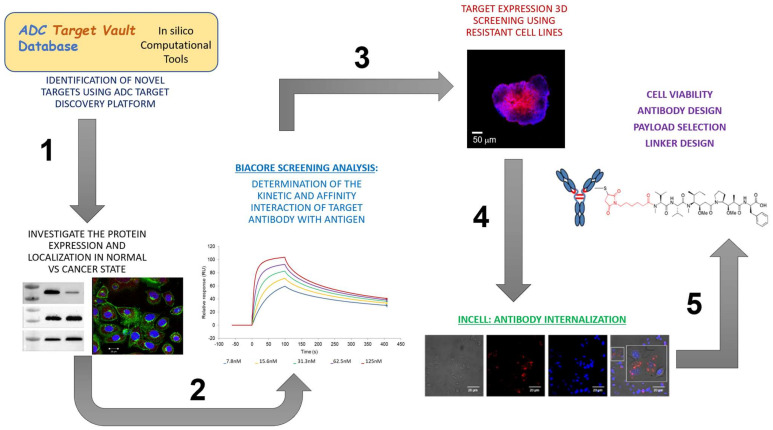
Schematic representation of the preliminary stages of target identification and validation prior to ADC conjugation phase.

In this study, we created an in silico database “ADC Target Vault” to identify potential targets for ADC development cancer. Our bespoke bioinformatic approach aggregated data from online sources to create this comprehensive database for querying potential ovarian cancer (OC) candidate targets suitable for ADC development. We successfully identified five targets overexpressed in ovarian cancer compared to healthy ovaries, with low or no expression in healthy tissues using a combination of publicly available data and our in vitro data. These candidates were then subjected to validation through in vitro pipelines before proceeding to the ADC conjugation phase (Figure 10). Our SPR platform was successfully applied to identified antibody–target pairs with good affinity and enhance internalisation capacity, validating the database target predictions. Additionally, the use of 3D cultures provided an insight into the target expression in vitro that could aid the design of in vivo cell line-derived xenograft (cdx) models to assess ADC efficacy and tumour penetration.

From the list of targets generated, we focused on five targets that were linked to biological processes which, when impaired, promote tumour progression and metastasis. From these targets, LRP6 is involved in the Wnt-signalling pathway, which is prominent in colorectal cancer, but has also been implicated in ovarian cancer. Additionally, the LRP6-mediated Wnt pathway is significant in ovarian cancer progression due to its ability to promote cancer stemness and metastasis, the latter being a major feature in ovarian cancer [39]. PCDHB10 and PCDHB15 are members of the cadherin superfamily of calcium-dependent cell–cell adhesion molecules; PCDHB10 is involved in the establishment and maintenance of neuronal functions in the brain and is implicated in paediatric malignancies [40]. PCDHB15 is involved in cell adhesion, and it is epigenetically regulated in melanoma and breast cancer cells [41,42]. Furthermore, OPRK1 has been implicated in facilitating the migration of breast cancer cells, while the low expression of GABRB1 correlated with a poor prognosis in colon adenocarcinoma [43,44]. None of these proteins have been previously identified as targets for ADC development. In addition to the bioinformatic approach adopted in this study to identify candidate targets, the use of ‘reverse immunology’, which involves the implementation of specific algorithms that predict the tumour-associated antigen (TAA) epitopes of the target receptors, thereby reducing the presence of extracellular loops, can be considered as a bioinformatic improvement.

### 4.1. Target Selection Significance

Identifying the optimal target represents the pivotal first step towards achieving success in ADC development. In this regard, in silico approaches such as the one described here for our ADC Target Vault database, that can discern and comprehensively profile potential antigen candidates, play a paramount role within the drug development process for these therapies. These targets should exhibit high expression in one or more tumour types while maintaining minimal expression in normal tissues. Achieving this balance remains a considerable challenge in ADC development.

The novel targets disclosed in this study are part of a larger database of proteins that could be targeted using ADC modalities. In particular, low-density lipoprotein receptor-related protein 6 (LRP6), an essential receptor for Wnt signaling, directly interacting with Wnt ligands, has been shown to be involved in multiple cancers [45]. Its extracellular domain also directly interacts with Dickkopf proteins to antagonize binding of Wnts and greatly reduces activity of Wnt-signalling pathways [46,47]. Hence, LPR6 targeting has been considered as a therapeutic option for cancer and neurodegeneration. The role of the Wnt/β-catenin pathway in ovarian cancer tumorigenesis, metastasis, immune evasion, and chemoresistance is well documented [48]. It has been shown that LRP6 silencing in ovarian cancer leads to a decrease in chemoresistance [39]. In this context, ADC targeting of LRP6 could be a promising approach in ovarian cancer but could also extend its translation as therapeutic for other cancer types.

Our computational approach enables the analysis of vast databases of biological information to predict potential and previously unknown target candidates. For instance, Protocadherin B10 (PCDHB10) was identified as potential ADC target based on its differential expression pattern. Despite its higher expression levels in multiple tumours’ biopsies (HPA data), this cell adhesion protein has usually been described as a tumour suppressor in colorectal, gastric, and pancreatic cancers [49]. Interestingly, it has also been shown to be upregulated in glioblastoma [50], suggesting PCDHB10 may associate with different proteins, depending on cell type to exert its action. Currently, there are no data regarding the effect of this protein on ovarian cancer proliferation, which could be important to further warrant its translation into ADC modalities.

PCDHB15, another protocadherin identified by us, is expressed in all ovarian cancer patient samples (Figure 2). However, similar to PCDHB10, it has been reported as a tumour suppressor. Nonetheless, like other cell adhesion proteins altered in specific cancer types, PCDHB15 can promote cancer cell proliferation and migration [41,42,43,51]. In vivo experiments have shown that PCDHB15 overexpression in melanoma promote cell invasion, cancer cell aggregation, and lung metastasis [43]. Interestingly, this gene was identified in an elegant study conducted by Schilling and colleagues as a predictor of platinum resistance. They employed a machine learning approach to identify biomarkers that can predict the outcomes of ovarian cancer patients and determine their platinum resistance status, leveraging publicly accessible gene expression data [52].

Although other databases like the one described by Schilling and colleagues filter and rank targets according to their gene expression profile, we decided not to follow this approach when selecting targets for ADC development. In fact, our mRNA data does not correlate to the higher levels of target protein observed in patient samples and cell lines. It is important to exercise caution when interpreting mRNA expression data, as there may be cases in which mRNA is not translated into protein or protein is produced but not presented on the cell surface. For example, LRP6 is a known target of microRNA (miR)-1271 in papillary thyroid carcinoma [53]. miRNAs mediate the degradation of mRNA target genes, but also regulate gene expression by regulating transcription and translation through canonical and non-canonical mechanisms. Another example is the post-transcriptional regulation of proto-cadherin 10 (PCDHB10) mRNA by miR-576-5p [54]. For this reason, we deliberately excluded RNA-seq data from our computational pipeline for target identification, and our approach was focused on analysing protein expression levels at the cell surface available for ADC binding.

A strict selection criterion was followed for the selection of the targets described here and those contained within our ADC Target Vault database. Ensuring specificity in target selection is critical for minimizing off-target toxicities and optimizing therapeutic efficacy. However, few antigens are truly tumour-specific with no expression in healthy tissue [55]. The vast diversity in tumour types and the heterogeneity within individual tumours further complicates ADC target selection, especially when targeting ovarian cancer, demanding the identification of antigens with broad relevance or tailored approaches for specific patient populations [55]. Here, we followed an approach where we identify targets based on their differential expression but also according to their expression in multiple cancer types, with those involved in multiple disease indications given priority in the ranking process.

One of the targets we identified as overexpressed in ovarian cancer was Gamma-aminobutyric acid type A receptor subunit beta1 (GABRB1). This protein is a metabotropic G-protein-coupled receptor that mediates the inhibitory effects of γ-aminobutyric acid (GABA) [44]. Although no in vivo or in vitro data have been reported for this protein in ovarian cancer, it has been shown that GABRB1is a prognostic biomarker of colon cancer and it is important contributor to prostate cancer [44,56]. Its inhibition of GABA signalling plays a crucial role in hepatocellular and pancreatic carcinomas [56]. Thus, suggesting this protein may be a therapeutic target in other cancers. Similarly, expression of OPRK1, an opioid receptor that belongs to the superfamily of G protein-coupled opioid receptors, has been reported to be associated with a significantly poorer prognosis and tumour migration in various cancers, such as breast, oesophageal squamous cell carcinoma, metastatic liver cancer, and pancreas neuroendocrine tumours [57,58]. A recent in vitro study conducted with OPRK1 antagonist and agonist identified OPRK1 as mediator of chloroxine therapy, suggesting this target could be further exploited in combination with other chemotherapies [59]. Although these two targets did not progress in our validation pipeline, this was mostly due to limitations around availability of commercial antibodies against these proteins.

Target validation and ongoing curation of our database will entail a comprehensive evaluation of the target’s expression levels in the target tumour, its accessibility to the antibody, and factors like internalization upon binding and target heterogeneity within the patient population. There is also increasing research into finding targets expressed in cells in the tumour microenvironment, particularly the stromal compartments not currently covered in our database [60]. Their inclusion into computational studies could further expand the antigen selection pool available for ADC development.

### 4.2. Challenges in ADC Development

ADCs continue to exhibit a relatively limited therapeutic range and have encountered challenges in achieving clinical success. The 10 main challenges in ADC development include target selection, antibody selection, linker design, payload selection, optimal conjugation chemistries, tumour heterogeneity, off-target toxicity, optimal pharmacokinetics, and pharmacodynamics (PK/PD) properties, overcoming resistance mechanisms and clinical translation of ADCs [60].

As the cornerstone of ADCs, the choice of target sets the stage for the entire development process, underscoring the need for meticulous evaluation. Additionally, antigen availability and accessibility can be limiting factors, constraining the pool of viable targets. In this context, in silico approaches are essential tools to accelerate target discovery and selection. Identifying the right target antigen that is highly expressed on tumour cells but minimally on normal cells is crucial. Here, we exemplified the power of our database using five targets that are minimally or not expressed in healthy tissue; however, not all our antibody–target pairs fulfil our selection criteria, with OPRK1 and GABRB1 failing to internalise bound antibodies (Figure 3 and Figure 4). Candidate-targeting antibodies must possess the immunogen of interest which binds to the extracellular region of the target antigen, facilitating cellular internalization, particularly in tumour cells [61,62]. Thus, this supports the premise of utilizing antibodies of the same target but with different epitopes, particularly in healthy tissue lysates, to identify target heterogeneity. A further issue in the selection of ADC targets is related to the homogeneity or heterogeneity expression patterns of the tumour marker on the tumour cell surface [61,63]. Homogenous expression of tumour targets has been demonstrated to favour ADC targeting more than those expressed heterogeneously [63]. Target distribution and expression within the tumour also needs to be investigated using screening methods that include the use of 3D spheroids, shown here, as they mimic cell–cell interactions between tumour cells [64]. Most importantly, since cellular spheroids possesses a higher degree of morphological and functional similarity to tissues, these models can be exploited to assess the tumour penetration efficiency of ADCs via a quantum dot-labelled antibody approach and monitored via fluorescence microscopy [61].

Selection of mAbs for ADC generation is dependent on binding stability and tumour penetrating ability [65]. Other factors include intracellular tracking of the ADC and epitope selectivity. For instance, studies have revealed that 0.001% to 0.01% of an injected unmodified antibody or an ADC, localizes to tumours in humans [66]. This limitation is due to the physiology and architecture of solid tumours, which contributes to the limited uptake of antibodies caused by the slow diffusion rates in poorly vascularized tumours. Furthermore, this restricted uptake is compromised by the fact that tumours often lack intratumour functional lymphatic vessels, which are restricted to the tumour margins and peritumoral regions. Although antibody–antigen interactions are required to facilitate cellular and tumour internalization, a balance between internalization and dissociation rates of antibody–antigen complexes is required to govern the effective delivery of the payload into the tumour space. However, this might not always be the case due to the nature of some target receptors possessing multiple spanning loops, which hinders antibody interaction as observed in this study. For instance, using SPR, we reveal poor interaction between anti-OPKR1-rOPKR1 and anti-GABRB1-rGABRB1, although antibodies still recognise the denatured recombinant proteins by immunoblot as well as the ovarian cancer samples (Supplementary Figure S4). This is in agreement with studies performed by Chu and colleagues, showing that challenges can occur using SPR to study multiple membrane spanning proteins [67].

The extent of internalization following binding to the target antigen may also facilitate bystander killing [68]. Here, we used SPR technology to select antibodies with good association constants (k_a_) to enhance target-mediated antibody internalisation. Interestingly, the antibody targeting LRP6 also exhibited a higher dissociation rate (high k_d_ value) while internalising to greater levels in ovarian cancer cells, which warrants future investigation. Antibodies that exhibit a higher off-rate at acidic pHs, mimicking the endosomal pH, can undergo dissociation from the receptor within the endosome, leading to the lysosomal trafficking of the ADC and release of the cytotoxic payload into the cell [69]. This is particularly important for receptors that are recycled back to the plasma membrane to reduce the likelihood that the ADC will cycle back out of the cell with its receptor [69]. Although we did not assess receptor recycling in our experiments, we did observe increased signalling of lysosomal location for our antibody pH–dye conjugates that exhibited a higher dissociation rate from the receptor. Our data corroborate the importance of dissociation kinetics, demonstrating a direct link between antibodies’ in vitro binding kinetics and internalization that can be exploited in identifying suitable candidates for ADC development and could be relevant when assessing antibody tumour penetration. For instance, several studies have reported that ADCs with a higher k_d_ also exhibited high penetration and distribution within solid tumours and increased efficacy, thus highlighting the importance of antibody affinity for ADC development [69]. Tsumura and colleagues investigated the effect of the dissociation rate constant on the intra-tumour distribution using anti-tissue factor (TF) ADCs with different k_d_ values [70]. Their data advocated for the selection of high affinity antibodies but also demonstrated that antibodies with higher k_d_ achieve better tumour penetration, homogenous distribution, and better efficacy in animal models. Rudnick et al. showed the effect of antibody affinity on antibody penetration on solid tumours by comparing two anti-human epidermal growth factor receptor 2 (HER2) IgGs trastuzumab (Herceptin^®^) and C6.5 (in IgG format, C6.5-IgG) [70]. They reported that C6.5 has a higher k_d_ than trastuzumab and this faster off rate allowed a higher penetration in solid tumours. ADCs must strike a balance between strong binding to the target antigen (high k_a_) to facilitate internalization and efficient release of the cytotoxic payload. Antibodies with appropriate k_d_ values can achieve this balance, ensuring that the payload is released within the tumour cell, leading to effective cell killing. Furthermore, Goldmacher and Kovtun have proposed that the initial assumption of a positive link between ADC target affinity and cytotoxicity is flawed, as ADCs with high target affinity strongly bind to the vascular structures surrounding the tumour rather than achieving uniform binding across all tumour cells [71]. Overall, these findings highlight the importance of SPR studies in ADC design, suggesting that both k_a_ and k_d_ are important when selecting therapeutic antibody modalities.

The interactions observed via SPR (in vitro binding) might not entirely translate to target-mediated internalization in vivo. Factors such as cell surface target expression need to also be considered. Moreover, the protein–receptor complex internalization rate relative to the normal receptor turnover rate can cause post-treatment down-regulation or upregulation of the receptor [72]. Further consideration regarding expression levels and antibody internalization properties of targets needs to be considered when selecting lead candidates. For instance, in tumour cells expressing high target expression, poor internalization rate may be compensated with the excess amounts of target on the cell surface to facilitate receptor-mediated ADC internalization. Therefore, for cells with low target expression, antibody-mediated internalization is crucial to compensate for reduced amounts of cell surface targets.

### 4.3. Clinical Implications

Although several therapies are approved as cancer treatments, from conventional to targeted therapies, including VEGF inhibitors, immunotherapies, and PARP inhibitors, appearance of chemoresistance hinders the therapeutic benefits, highlighting the need to develop biomarker-based therapies to address these challenges [2,3,4,5,6]. Ovarian cancer remains a challenging disease to treat, especially for patients that progress on these treatments. Its features include tumour heterogeneity, poor prognosis, and recurrence due to chemotherapy resistance mostly associated with germline BRCA1/2 mutations [13].

Mirvetuximab soravtansine is the first and only FDA-approved ADC for ovarian cancer [73]. The SORAYA study, a phase III clinical trial, evaluated the clinical activity of this ADC in patients with FRα-positive (IHC score +2) platinum-resistant high-grade serous epithelial ovarian who had received up to three prior lines of therapy [74]. Overall, the study reported that 32% of patients achieved objective responses, with 5 (5%) complete responses and 29 (28%) partial responses [74]. There are multiple ongoing clinical trials evaluating ADCs in ovarian cancer, including those that target TROP2, mesothelin, and HER2 [55].

Approximately 60% of serous epithelial ovarian cancer has high FRα overexpression [73]. Identifying targets with similar or better coverage among the patient population and able to overcome the inherent heterogeneity of ovarian cancer remains a challenge. Among the targets identified here, PCHDB15 was homogeneously expressed in 100% of patient samples, followed by LRP6, with more than 90% of tumours expressing the target in more than 75% of ovarian cancer cells. Similarly, 83% of tumours expressed PDCHB10 in more than 75% of ovarian cancer cells, whereas this distribution of staining was observed in 75% of tumours assessed for GABRB1 expression. In the case of OPRK1, only 50% of tumours expressed this antigen in more than 75% of cells (Figure 2), but its expression profile against healthy tissue was excellent compared to other targets, including FRα. Other targets selected form our database and in development in our lab exhibited similar distribution within the ovarian cancer tumours and against healthy tissue. Notably, all our targets were expressed in cisplatin-resistant cells, which further address an area of unmet clinical need in ovarian cancer. The clinical implications of the database as a tool for ADC development will be realised once these targets are validated using larger number of patient samples, in vivo patient-derived xenograft models, and clinical trials. Benchmarking these novel targets against approved ADCs, including FRα, HER2, Nectin 4, and TROP2, will aid the identification of lead therapeutic candidates for treatment of solid tumours [55].

### 4.4. Limitations of the Study and Future Research

One of the main limitations of our study includes the availability of commercial antibodies and recombinant proteins for validation. For example, we did not observe any internalization with the antibodies selected for the OPRK1 and GABRB1 targets, but these should not rule out these targets, and future efforts should concentrate on developing antibody libraries against these proteins with excellent expression profiles. Similarly, our database identified novel targets for which experimental tools such as recombinant proteins are yet to be available. Another limitation is the availability of patient samples to assess for the in vitro antibody–target internalisation experiments. Finally, we were unable to assess the conjugate versions of the antibodies tested in this study. Future research efforts will be focused on validating more targets, assessing the potential of the ADC conjugates in multiple cancer types, and making the ADC Target Vault database publicly available as a resource to accelerate drug development in ovarian cancer as well as in other cancer types.

## 5. Conclusions

In this study, we present a bioinformatic pipeline approach for target identification and antibody selection, which are pivotal in ADC development (Figure 10). Beyond elucidating crucial criteria for target selection, such as significantly higher expression in healthy cells than in cancer cells, cell surface immunogenicity, robust antibody–antigen binding, and the capacity for antibody internalization, we underscore the value of dynamic antibody–antigen interaction assessed through SPR. This dynamic insight holds promise in predicting antibody internalization within 2D models and tumour penetration in 3D cancer cell models.

Of paramount significance, we identify three promising ovarian cancer targets suitable for ADC development: LRP6, PCDHB10, and PCDHB15. The application of the methodologies described in this study will allow for better understanding of desirable properties of ADC targets to overcome current limitations in ADC development and ultimately aid discovery of new ADC targets, patient selection, and better clinical outcomes.

## Data Availability

All related data and methods are presented in this paper. Additional inquiries should be addressed to the corresponding author.

## Supplementary Materials

The following supporting information can be downloaded at: https://www.mdpi.com/article/10.3390/antib12040065/s1, Figure S1: ADC Target Vault Database Schema; Figure S2: (A) Controls for immunofluorescence assay using monolayers. Ovarian cancer cell lines were cultured as 2D monolayers in 8 well chamber slides and stained with nuclei marker (DAPI) and secondary antibody. Magnification: 40×, Scale bars: 20 μm. (B) Quantitative analysis of target fluorescence intensity in OVCAR3 WT cells was determined using ImageJ. Data are represented as a mean of 40 individual cells under different conditions. Data are +/− SD. Figure S3: SKOV3 spheroid formation and immunofluorescence controls (A) Representative brightfield micrographs of SKOV-3 derived spheroids for 24–72 h. Spheroid formation was observed using live cell microscopy. Cells aggregated into a spheroid-like structure 24 h post-culture and became more compact with clear boundaries at 72 h. (B) quantification of brightfield images. (C) SKOV3 spheroids were grown via liquid overlay method and stained with nuclei marker (DAPI) and secondary antibody. Magnification: 40×, Scale bars: 20 μm. Figure S4: Internalisation controls and Recombinant protein detection. (A) OVCAR3 WT and cisplatin resistant cell lines were cultured as 2D monolayers and treated for 4 h with pH sensitive dye only. Cells were stained with nuclei marker (DAPI) and secondary antibody. Magnification: 40×, Scale bars: 20 μm. Specificity of OPKR1 and GABRB1 antibodies was tested using 15 ng of the targets recombinant proteins (B) rOPKR1 & (C) rGABRB1 respectively and equal amount of recombinant rCDH20 protein (-ve), used as a negative control. Table S1: Description of the data sources which we used to create the “ADC Target Vault” database. Table S2: List of tables that are in the ADC Target Vault database, together with a description of the data that each table contains; Table S3: List of antibodies used for the current study. Table S4: Kinetics and general data for LRP6, PCDHB10 and PCDHB15 targets.
