# Supplementary material for: Elucidating Novel Targets for Ovarian Cancer Antibody–Drug Conjugate Development: Integrating In Silico Prediction and Surface Plasmon Resonance to Identify Targets with Enhanced Antibody Internalization Capacity
Source: Antibodies (Basel). 2023 Oct 16;12(4):65. doi: 10.3390/antib12040065 (PMC10594448; doi:10.3390/antib12040065)
Supplement: Supplementary file 1 [file antibodies-12-00065-s001.zip › antibodies-2620246-supplementary.pdf]

Figure S1

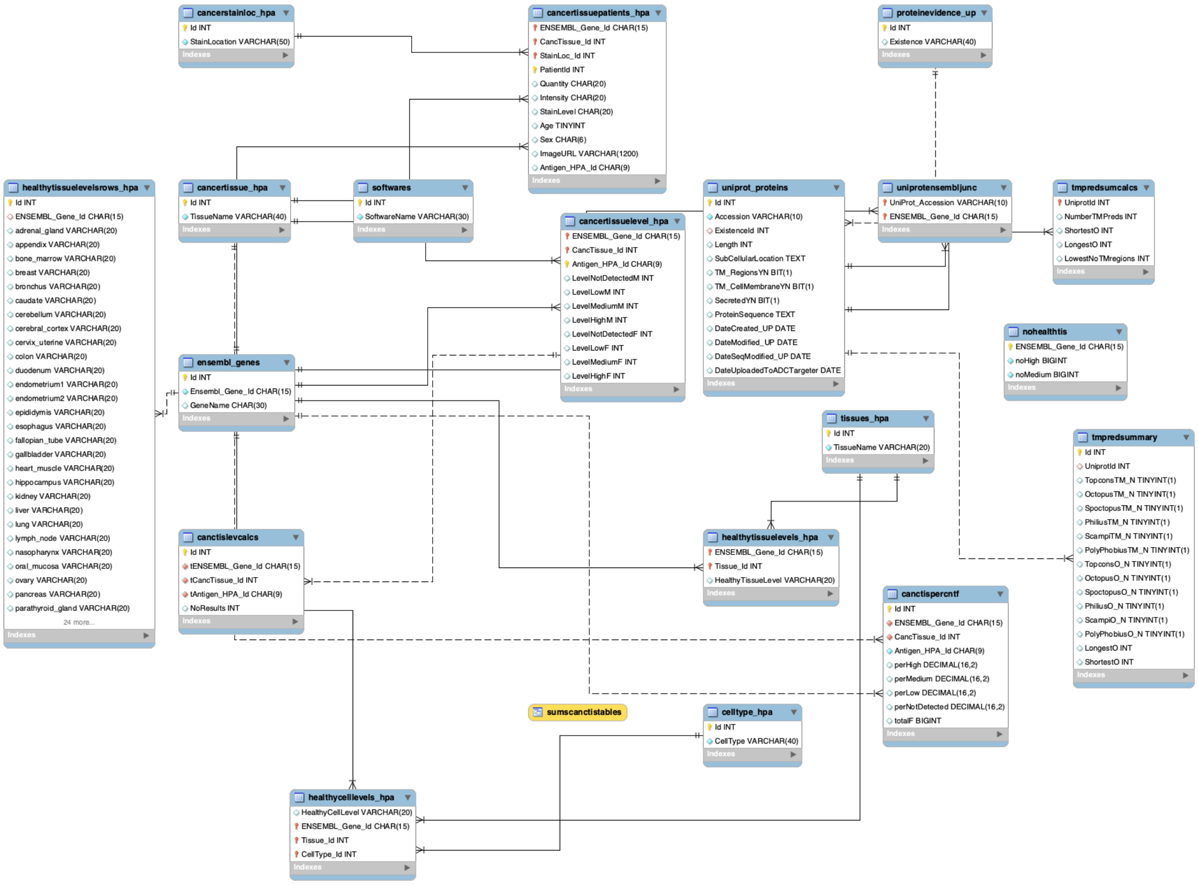

Figure S1. ADC Target Vault Database Schema

This is a diagram of the database schema used in the study. The following link connects to its GitHub page https://github.com/RBGOLab/ADC-database-generation-scripts (link created on 15th of September 2023) .

Figure S2.

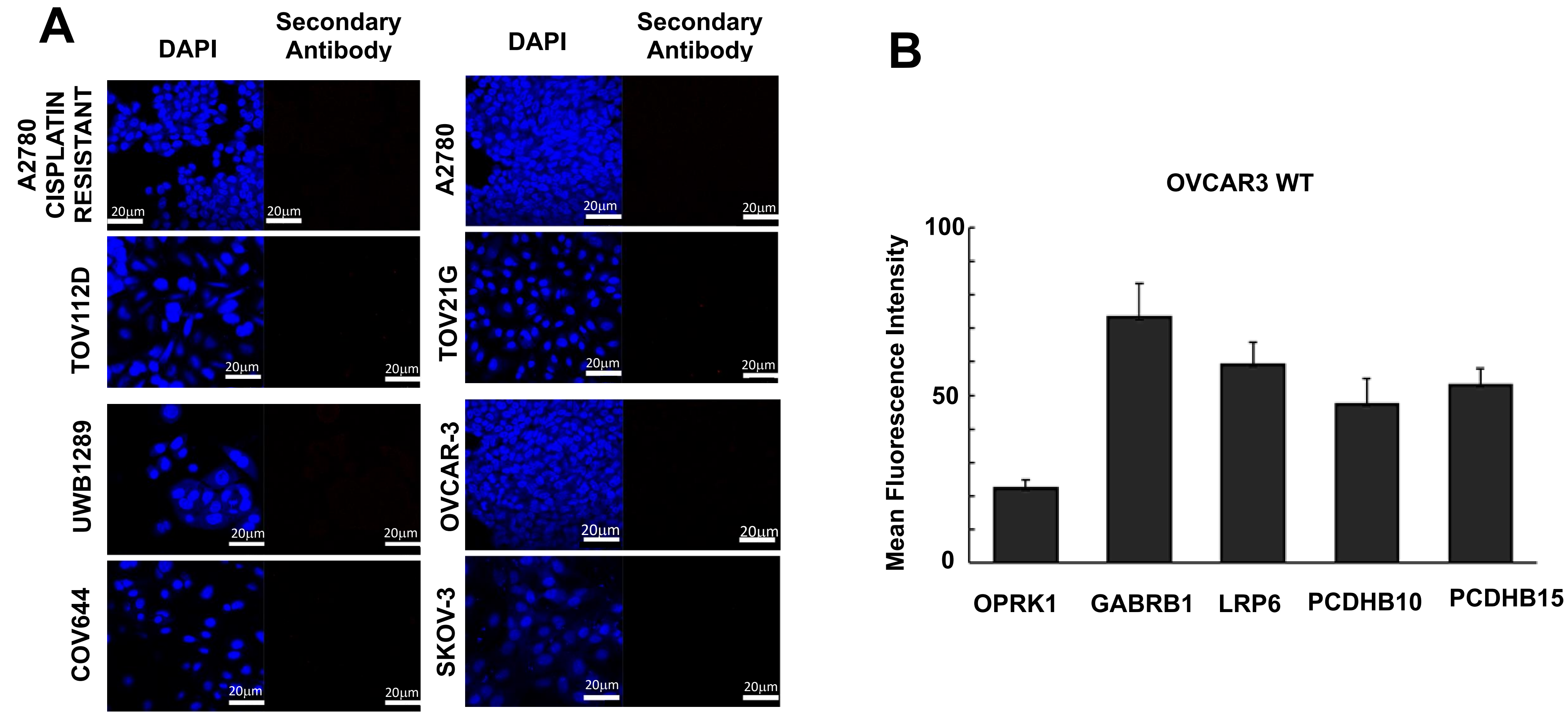

Figure S2: A) Controls for immunofluorescence assay using monolayers. Ovarian cancer cell lines were cultured as 2D monolayers in 8 well chamber slides and stained with nuclei marker (DAPI) and secondary antibody. Magnification: 40×, Scale bars: 20 μm. **B)** Quantitative analysis of target fluorescence intensity in OVCAR3 WT cells was determined using ImageJ. Data are represented as a mean of 40 individual cells under different conditions. Data are +/- SD.

Figure S3.

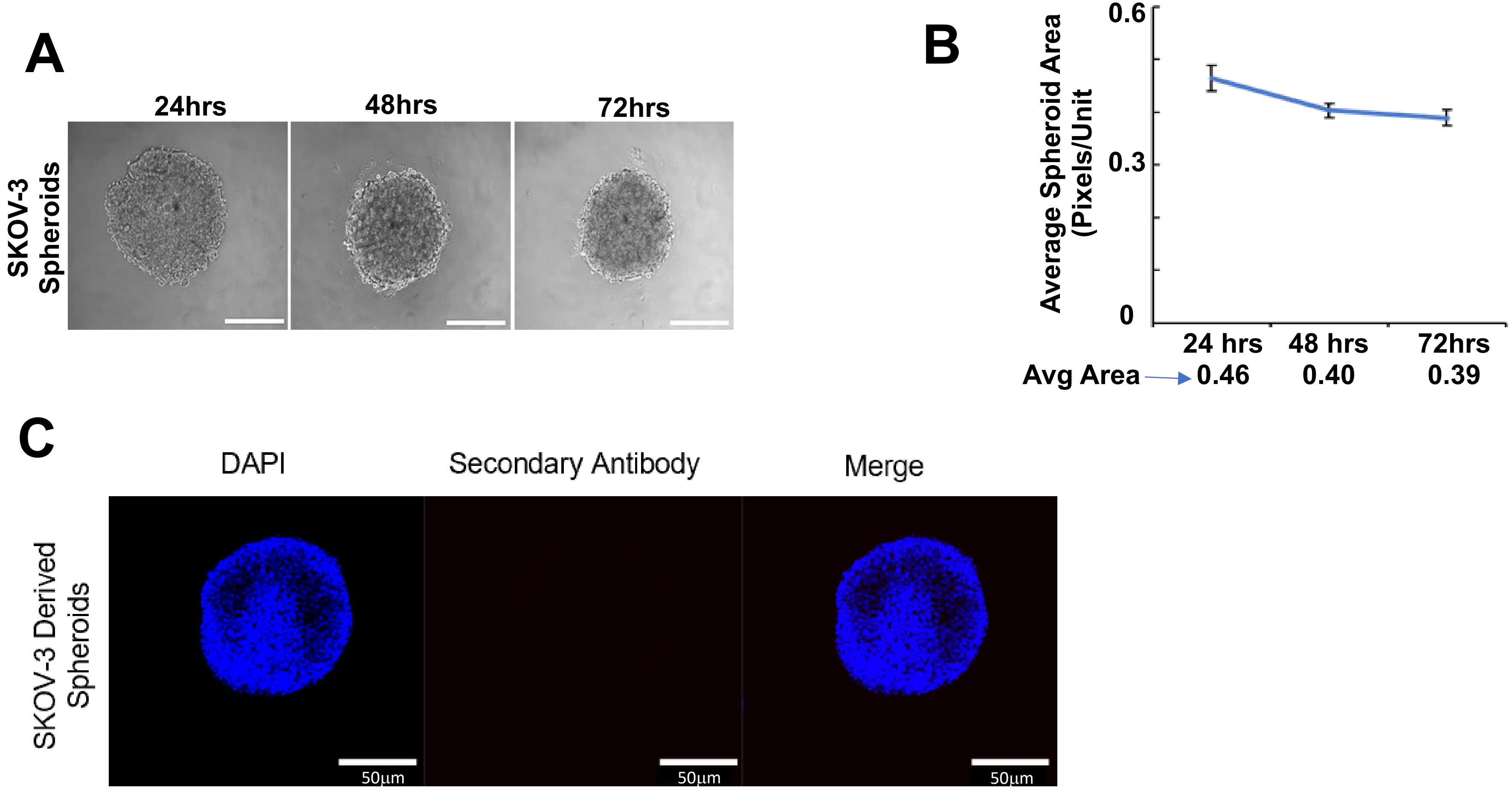

Figure S3: SKOV3 spheroid formation and immunofluorescence controls **A)** Representative brightfield micrographs of SKOV-3 derived spheroids for 24 – 72 hours. Spheroid formation was observed using live cell microscopy. Cells aggregated into a spheroid-like structure 24hrs post-culture and became more compact with clear boundaries at 72 hrs. **B)** quantification of brightfield images. **C)** SKOV3 spheroids were grown via liquid overlay method and stained with nuclei marker (DAPI) and secondary antibody. Magnification: 40×, Scale bars: 50 µm.

Figure S4.

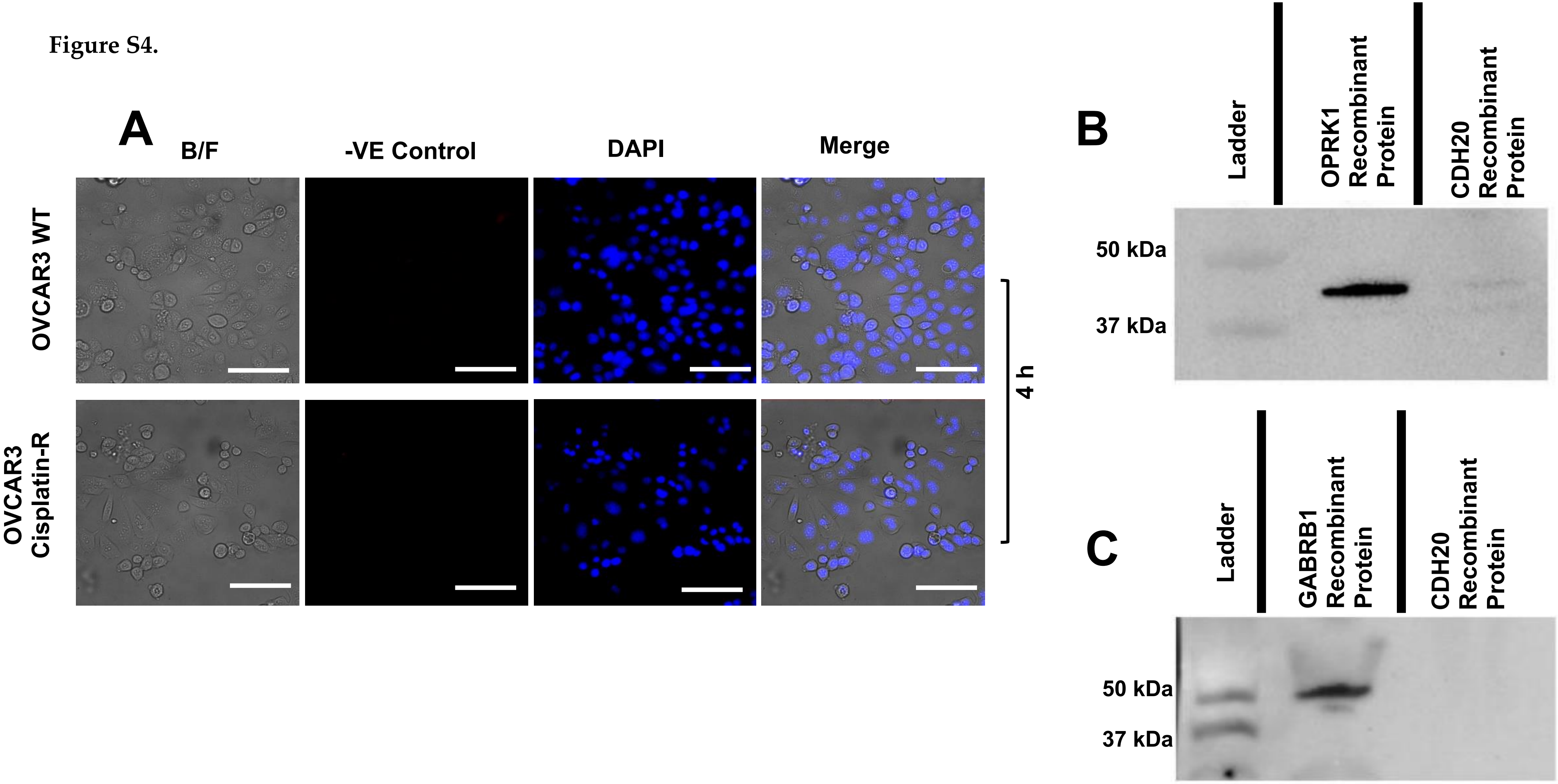

Figure S4: Internalisation controls and Recombinant protein detection. **A)** OVCAR3 WT and cisplatin resistant cell lines were cultured as 2D monolayers and treated for 4h with pH sensitive dye only. Cells were stained with nuclei marker (DAPI) and secondary antibody. Magnification: 40×, Scale bars: 20 µm. Specificity of OPRK1 and GABRB1 antibodies was tested using 15ng of the targets recombinant proteins **B)** rOPRK1 & **C)** rGABRB1 respectively and equal amounts of recombinant rCDH20 protein (-ve), used as a negative control.

Supplementary Table S1

Description of the data sources which we used to create the "ADC Target Vault" database

Database	URL	Description
ENSEMBL	https://www.ensembl.org (accessed 12.11.2018)	Genome browser for vertebrate genomes with annotations
UniProt	https://www.uniprot.org/ (accessed 12.11.2018)	Database of protein sequences and functional information
Human Protein Atlas	https://www.proteinatlas.org/ (accessed 12.11.2018)	Database mapping proteins in cells, tissues and organs from various omics, imaging and mass-spec technologies for healthy and disease states
TopCon	https://topcons.cbr.su.se/ (accessed 28/01.2019)	Webserver for consensus transmembrane prediction results from 6 different computational tools

Table S2
List of tables that are in the ADC Target Vault database, together with a description of the data that each table contains.

**Table**	**Description**
cancerstainloc_hpa	Cellular location of cells from HPA
cancertissue_hpa	List of cancer tissues in the HPA
cancertissuelevel_hpa	Summary table with number of patients with low/medium/high levels of stain from IHC images from HPA
cancertissuepatients_hpa	Patient wise IHC image data from HPA
canctislevcalcs	Levels available for cancer tissue from IHC images
canctispercntf	Gene wise percentage of cancer IHC images with stain level
celltype_hpa	List of cell types from HPA
ensembl_genes	ENSEMBL genes and gene symbols
healthycelllevels_hpa	Protein expression levels in healthy cells from HPA
healthytissuelevels_hpa	Protein expression levels in tissue from HPA
healthytissuelevelsrows_hpa	Swap rows and columns from healthytissuelevels_hpa table
nohealthtis	Gene wise number of tissues with high or medium expression levels in healthy tissues from HPA
proteinevidence_up	Levels available for protein evidence in UniProt
softwares	List of transmembrane prediction tools used
tissues_hpa	Tissues available in HPA
tmpredsumcalcs	Protein wise summary of transmembrane predictions from transmembrane prediction tools
tmpredsummary	Protein wise summary of transmembrane
uniprot_proteins	Protein data from Uniprot
uniprotensembljun	Junction table for linking UniProt IDs with ENSEMBL gene IDs

Table S3: List of antibodies used for the current study.

Primary Antibody	Manufacturer	Ab Type	Antibody Dilutions			
			SPR	Western Blot	Confocal	InCell 6000
**Anti-LRP6 (N-Terminus)**	Sigma-Aldrich	Rabbit Polyclonal	1:1000	1:1000	1:50	1:50
**Anti-PCDHB10 (N-Terminus)**	Sigma-Aldrich	Rabbit Polyclonal	1:1000	1:1000	1:50	1:50
**Anti-PCDHB15 (N-Terminus)**	Sigma-Aldrich	Rabbit Polyclonal	1:1000	1:1000	1:50	1:50
**Anti-LRP6 (C-Terminus)**	Sigma-Aldrich	Rabbit Polyclonal	N/A	1:1000	N/A	N/A
**Anti-PCDHB10 (C-Terminus)**	VWR	Rabbit Polyclonal	N/A	1:1000	N/A	N/A
**Anti-PCDHB15 (C-Terminus)**	Sigma-Aldrich	Rabbit Polyclonal	N/A	1:1000	N/A	N/A
**Anti-OPRK1 (N-Terminus)**	Abcam	Mouse Polyclonal	N/A	1:1000	N/A	N/A
**Anti-GABRB1 (N-Terminus)**	Origene	Rabbit Polyclonal	N/A	1:1000	N/A	N/A
**Anti-OPRK1 (C-Terminus)**	Abcam	Rabbit Polyclonal	N/A	1:1000	N/A	N/A
**Anti-GABRB1 (C-Terminus)**	Abcam	Mouse Monoclonal	N/A	1:1000	N/A	N/A

**Wheat Germ Agglutinin, Alexa Fluor™ 488 Conjugate (W11261)**	ThermoFisher	N/A	N/A	N/A	1:700	1:700
**Wheat Germ Agglutinin, Alexa Fluor™ 594 Conjugate (W11262)**	ThermoFisher	N/A	N/A	N/A	1:700	1:700
**Cross-absorbed secondary antibody, Alexa Fluor 594, A11012**	ThermoFisher	Goat anti-Rabbit IgG (H&L)		N/A		1:400
**Alexa Fluor 488, A11001**	ThermoFisher	Goat anti-Mouse IgG (H&L)		N/A		1:400

Table S4. Kinetics and general data for LRP6, PCDHB10 and PCDHB15 targets.

TARGET	SPR ANALYSIS (ANTIBODY-ANTIGEN) OFF-RATE SCREENING	PROTEIN LOCALIZATION USING WGA	PROTEIN EXPRESSION IN OVARIAN CANCER CELL LINES	BIOLOGICAL PROCESS	BLOOD SPECIFICITY (HPA)	TISSUE SPECIFICITY	EXPRESSION IN OTHER CANCER TYPES (HPA)
**LRP6**	k_d=3.98 x 10^{-4} 1/s<br>k_a=3.68 x 10^{5} 1/s<br>K_D=1.08 x 10^{-9} 1/s	Membrane/ Cytoplasmic	TOV21G, A2780 (cisplatin resistant)	Wnt Signalling endocytosis	Naive CD4 T-cells	Low tissue specificity	Ovarian<br>Renal<br>Colorectal<br>Liver<br>Endometrial<br>Prostate<br>Breast
**PCDHB10**	k_d=7.19 x 10^{-5} 1/s<br>k_a=3.64 x 10^{5} 1/s<br>K_D=1.98 x 10^{-10} 1/s	Membrane/ Cytoplasmic	A2780 (cisplatin resistant), UWB1289 UACC1598	Involved in the establishment of specific neural connections in the brain	Not detected in immune cells	Low tissue specificity	Liver<br>Testicular<br>Pancreatic<br>Endometrial<br>Urothelial
**PCDHB15**	k_d=8.23 x 10^{-5} 1/s<br>k_a=1.12 x 10^{5} 1/s<br>K_D=7.34 x 10^{-10} 1/s	Membrane/ Cytoplasmic	A2780 (wild type and cisplatin resistant), OVCAR3, UACC1598	Cell adhesion, establishment of specific neural connections in the brain	Memory CD8 T-cells	Low tissue specificity	Endometrial<br>Ovarian,<br>Melanoma,<br>Liver,<br>Breast,<br>Colorectal,<br>Pancreatic
